# Systematic Investigation on Acid-Catalyzed Truncation of *N*-Acylated Peptoids

**DOI:** 10.3390/ijms252111390

**Published:** 2024-10-23

**Authors:** Ruiqi Piao, Yong-Uk Kwon

**Affiliations:** Department of Chemistry and Nanoscience, Ewha Womans University, Seoul 03760, Republic of Korea; piaoruiqi@163.com

**Keywords:** amide bond cleavage, conformational effect, electronic effect, *N*-acylated peptoid, *n*→π* interaction

## Abstract

Peptoids have emerged as a useful alternative to peptides. However, *N*-acylated peptoids have occasionally undergone truncation at the terminal peptoid unit under acidic conditions. We previously reported on the mechanistic and electronic aspects of the acid-catalyzed truncation of *N*-acylated peptoids. To gain further insight, we systematically investigated the conformational and electronic effects of the terminal side chains of peptoids. The *n*→π* interaction, based on *cis/trans*-amide bond conformation, is considered to be one of the determining factors. In this study, it was demonstrated that both conformational and electronic factors contribute to this unusual truncation.

## 1. Introduction

Peptoids (*N*-substituted glycine oligomers) within the family of peptidomimetics have garnered increasing interests from researchers [1,2,3,4]. The absence of amide hydrogen in their backbone structure endows them with diverse structural and functional features, such as secondary structures [5], improved cell permeability [6], and resistance to proteolysis [7]. Over the past decades, the range and utility of peptoids have expanded to include numerous functions, such as asymmetric catalysis [8], molecular transport [9,10], nanomaterial properties [5,11], and disease diagnosis [12]. Peptoids are also gaining popularity in the field of therapeutics [13,14]. This wide range of applications highlights the interest among chemists and biologists in exploring the diverse potential of peptoids.

Edman degradation is widely used for the sequential degradation of polypeptides, utilizing the reaction with phenylisothiocyanate followed by treatment with trifluoroacetic acid. This iterative degradation method can also be successfully applied to peptoids [15]. Additionally, several reports have indicated that *N*-acetylated peptides with *N*-methylamino acid at the *N*-terminal position can be truncated under acidic conditions [16,17]. Similar truncation has been observed in *N*-acylated amino acid amides or peptides, demonstrating acyl group-dependent instability under acidic conditions [18]. In that study, it was shown that the lability of dipeptide is correlated with the Hammett σ constant of benzoyl substituents. Recently, a mild approach for the *N*-terminal degradation of peptoids, involving bromoacetylation followed by treatment with silver perchlorate, was reported [19].

**Figure 1:**
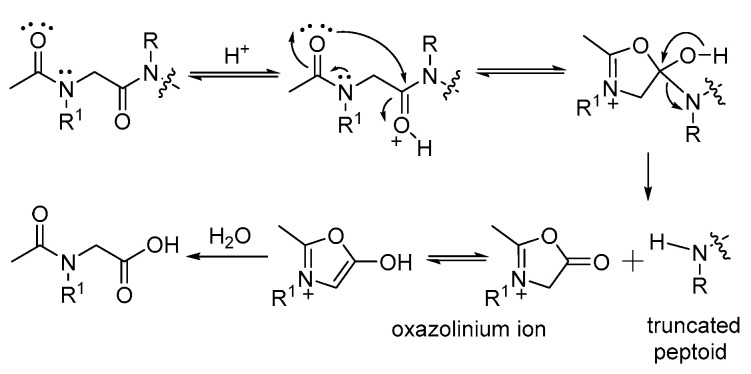
Proposed mechanism for the *N*-terminal truncation of *N*-acetylated peptoid [20].

As a method for the *N*-terminal degradation of peptoids, we previously reported the truncation of *N*-acylated peptoids under acidic conditions [20]. In this study, the electronic effects of acyl groups were thoroughly investigated to support the proposed truncation mechanism (Figure 1). The inclusion of strong electron-withdrawing functionalities, such as 4-nitrobenzoyl and trifluoroacetyl groups, was found to reduce the truncation effect compared to the introduction of electron-donating groups.

These results motivated us to explore the structural perturbation caused by *N*-alkyl side chains in the acid-mediated truncation of *N*-acylated peptoids. We also aimed to investigate whether the deletion of the *N*-terminal unit in peptoids is influenced by both the conformation and the electronic effects of the *N*-α branching side chain. In this study, we present a detailed systematic analysis of the role of *N*-terminal side chains in determining the unusual truncation of *N*-acylated peptoids.

## 2. Results and Discussion

In our previous studies, we reported that when the isopropyl group was present as a terminal side chain, the truncated products were the only ones observed [20]. The *cis/trans*-amide isomerism of peptoids is also known to be influenced by the incorporation of branched side chains with steric and stereoelectronic characteristics [21]. This result led us to hypothesize that the conformational effect of side chains plays a significant role in this peptoid truncation. To test this hypothesis, we examined other variants of the isopropyl group, including *tert*-butyl, 2-butyl, cyclohexyl, and cyclopentyl groups. Using the conventional submonomer strategy for peptoids synthesis, we synthesized several model peptoids containing these branched alkyl side chains to facilitate comparisons (Table 1). We found that peptoids (**1A′**–**4A’**) produced the truncated peptoid **6** quantitatively after treatment with 50% trifluoroacetic acid (TFA) for 1 h. Peptoid **5A′** was converted to the truncated peptoid **6** with a 93.4% yield after 1 h and a quantitative yield after 3 h. These experimental data (Supplementary Materials: Table S1 and Figures S1–S8) aligned well with our initial assumptions, clearly demonstrating that the conformational effects of branched side chains at the terminal units play a key role in this acid-catalyzed degradation.

To investigate the influence of chain length and sequence on truncation, we also treated peptoids of various chain lengths (4 mer–7 mer) containing an *N*-cyclohexyl group as the terminal side chain under acidic conditions (Table 2). Three peptoids (entries 1–3) gave the corresponding truncated peptoids as the only products after a 1 h reaction in both 50% and 92% TFA. The degradation of the longer peptoid **9A′** was not complete after 1 h in either TFA solution. However, it afforded the truncated product in quantitative yield after 3 h under both conditions. Additionally, peptoid **10A′** with a different sequence produced about 74% and 100% of the truncated peptoid **11** after treatment with both TFA cocktails for 1 h and 3 h, respectively. These experiments (Table S2 and Figures S9–S19) demonstrated that the truncation of *N*-acetylated peptoids with a branched cyclohexyl side chain is not significantly dependent on the chain length or sequence of the peptoids.

To determine whether the unusual truncation reaction also occurs in the solution phase, we prepared peptoid **4A′** on TentaGel MB RAM resin, buffered by photo-cleavable ANP linker (Scheme S1 and Figures S20–S21) [22]. The *N*-acetylated linear peptoid was then cleaved from the resin by UV irradiation in a MeOH/H_2_O (1/1) mixture for 3 h, yielding the pure non-truncated sequence (**4A**), which was collected from HPLC and then lyophilized. The compound (**4A**) was subsequently treated with a 92% TFA cocktail mixture in solution phase, resulting in the quantitative formation of the truncated peptoid (**6**). In contrast, treating the pure *N*-acetylated peptoid (**4A**) with a different acid cocktail of HCl (12 N)/MeOH (1/1) for 1 h yielded only 37.8% of the truncated peptoid.

To elucidate the influence of *N*-aryl side chains on acid-assisted truncation of *N*-acetylated peptoids, we synthesized several peptoids with *N*-aryl groups at the terminal position (Table 3). We employed four aniline derivatives—aniline, *p*-anisidine, ethyl 4-aminobenzoate, and 4-nitroaniline—as terminal side chains. Due to their lower reactivity, the aromatic amines were first incubated with the resins for 12 s in a microwave, repeated three times, followed by shaking the reaction mixture at 40 °C for 4 h. Three of the amines, except 4-nitroaniline, which did not react even at elevated temperatures, underwent nucleophilic substitution to afford the corresponding linear peptoids containing a terminal *N*-aryl side chain. The unprotected terminal secondary amines of these peptoids were then capped with acetic anhydride (50 eq.), *N,N*-diisopropylethylamine (DIPEA) (50 eq.), and 4-dimethylaminopyridine (DMAP) (10 eq.) at 35 °C overnight. This treatment was repeated to achieve the complete conversion of the unprotected terminal amines to *N*-acetylated peptoids (**12A′** and **13A′**). For the peptoid containing 4-aminoethylbenzoate, the resins were treated twice with acetyl chloride (50 eq.) and DIPEA (50 eq.) in THF 35 °C overnight, yielding the *N*-acetylated peptoid (**14A′**).

The *N*-acetylated peptoids (**12A′**–**14A′**) were treated with a cleavage cocktail of 50% or 92% TFA for both 1 h and 3 h. As expected, the degradation was significantly slowed by the electronic effects of the aryl side chains. Additionally, the differences in truncation efficiency corresponded with the p*K*a values of the conjugate acids of the aryl amines. Specifically, in the case of *p*-anisidine (entries 3–4), which contains an electron-donating group, the deletion sequence was produced slightly more than with aniline. Conversely, in the case of ethyl 4-aminobenzoate (entries 5–6), which contains an electron-withdrawing group, the corresponding deletion sequence was not detected. These results (Table S3 and Figures S22–S29) clearly indicate that the electron availability on the amide nitrogen is a key factor in this truncation, which is consistent with the proposed mechanism.

We also synthesized several peptoids with different chain lengths (4 mer–7 mer) and sequences, each containing an *N*-aryl side chain at the terminal unit, to determine whether these factors had any adverse effect on truncation (Table 4). In all cases (entries 1–4), the *N*-acetylated peptoids yielded the corresponding deletion products (**11**, **15B**–**17B**) (Table S4 and Figures S30–S38). Based on this observation, we can infer that chain length and sequence identity have little to no effect on the acid-catalyzed truncation.

Finally, to compare the effects of *N*-branched and *N*-unbranched alkyl side chains on relative truncation reactivity, we selected several alkyl side chains, including 2-butyl, isobutyl, n-butyl, and 1-phenylethyl groups, as terminal *N*-alkyl substituents. As expected, we observed the incomplete truncation of the peptoids (**18A′** and **19A′**) containing *N*-unbranched alkyl side chains, even after extended treatment with the acid mixture. In contrast, peptoid **3A′**, with a 2-butyl group, was completely truncated after 1 h (Table 5). The 1-phenylethyl group is noteworthy in peptoid chemistry due to its chiral nature, which can influence peptoid secondary structures. We observed that peptoid **20A′** was completely degradaded after treatment with 92% TFA for 3 h. It exhibited enhanced reactivity compared to the other peptoids (**18A′** and **19A′**) with *N*-unbranched side chains, but slightly lower reactivity than peptoid **3A′** with an *N*-branched side chain (Table S5 and Figures S39–S47).

**Figure 2:**
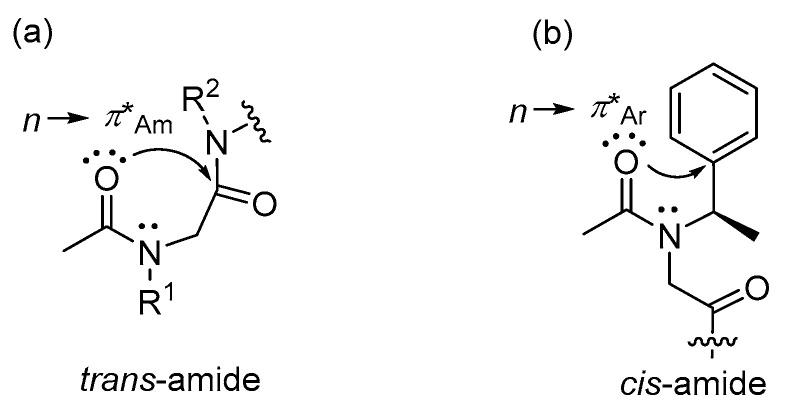
Schematic representation of (**a**) *n*→π*_Am_ and (**b**) *n*→π*_Ar_ interactions in mechanistic pathway.

These results may be influenced by several factors, including electronic and conformational effects. In particular, the *cis/trans*-amide isomerism of peptoid is likely critical in acid-catalyzed truncation. This isomerism may be regulated by *n*→π*_Am_ and *n*→π*_Ar_ interactions, where *n* represents a nonbonding orbital of amide oxygen, and π*_Am_ and π*_Ar_ are π* antibonding orbitals of the amide carbon and the aromatic ring at the side chain, respectively (Figure 2) [23]. The typical secondary structures of peptoids can be controlled by selecting appropriate side chains based on *cis/trans*-amide isomerism [24]. We hypothesized, based on the reaction mechanism pathway, that the *n*→π*_Am_ interaction facilitates the formation of a five-membered intermediate, leading to sequence deletion. Our study has shown that the *trans*-amide conformation favors peptoid truncation. Branched side chains as bulky electron-donating groups might enhance the *n*→π*_Am_ interaction in the *trans*-amide conformation due to both electronic and conformational effects. In the case of the 1-phenylethyl group, *cis*-amide conformation may occur via the *n*→π*_Ar_ interaction, causing the reaction to proceed more slowly due to the electron-withdrawing effect of the aromatic ring.

## 3. Materials and Methods

Chemical reagents were obtained from commercial suppliers and used without further purification unless otherwise specified. Rink amide AM (capacity: 0.37 mmol/g) and TentaGel MB RAM (capacity: 0.41 mmol/g) resins were purchased from GL Biochem and RAPP polymere, respectively. Peptoid synthesis was carried out using standard glass peptide synthesis vessels in an incubator shaker (JEIO TECH: Daejeon, Korea, model: SI-600R) or in a microwave oven (Daewoo: Seoul, Korea, model: KR-B200R) at a power of 100 W. Reverse-phase HPLC experiments were conducted using an ACE 5 C18-HL (250 mm × 4.6 mm) reverse phase column on a Shimadzu binary HPLC system equipped with a UV-visible detector set to 220 nm. MALDI-TOF MS was performed on an Axima Performance mass spectrometer (Shimadzu: Tokyo, Japan) using α-cyano-4-hydroxycinnamic acid as the matrix at the National Research Facilities and Equipment Center (NanoBio·Energy Materials Center) at Ewha Womans University.

### 3.1. General Procedure for Peptoid Synthesis

Peptoids were synthesized on Rink amide AM resins using the conventional sub-monomer strategy in 25 mL standard glass peptide synthesis vessels [2]. The resins were swollen in DMF at 25 °C for 1 h. After draining DMF, the beads were incubated with 20% piperidine in DMF for 1 h and thoroughly washed with DMF (8 × 3 mL). The beads were then treated with 2 M bromoacetic acid (2 mL) and 3.2 M DIC (2 mL) and irradiated in a microwave oven (100 W) for three 12 s intervals, with shaking for 30 s after each pulse. After thorough washing with DMF (8 × 3 mL), the beads were treated with primary amines (2 M, 2 mL) in DMF and irradiated in the microwave for three 12 s intervals, with shaking for 30 s after each pulse. This process of bromoacetylation followed by displacement was repeated to achieve the desired peptoid chain length.

For branched amines, the beads were treated with primary branched amines (2 M, 2 mL) in DMF and irradiated in the microwave (100 W) for three 12 s intervals, with shaking for 30 s after each pulse, followed by shaking the resins for 30 min at 25 °C in the incubator shaker.

For aromatic amines, the beads were treated with primary aromatic amines (2 M, 2 mL) in DMF in the microwave (100 W) for three 12 s intervals, with shaking for 30 s after each pulse, and then the resins were shaken 4 h at 40 °C in the incubator shaker.

### 3.2. General Procedure for Acetylation of N-Terminal of Peptoids

For the synthesis of *N*-acetylated peptoids, the resins containing the desired peptoid sequences were treated with acetic anhydride (50 eq.), DIPEA (50 eq.), and 4-dimethylaminopyridine (DMAP) (10 eq.) in DMF (2 mL) and shaken at 35 °C overnight. After this treatment, the resins were washed with DMF (8 × 3 mL) and DCM (8 × 3 mL). For peptoids with aromatic side chains at the terminal position, this treatment was repeated to ensure the complete conversion of unprotected terminal amines to *N*-acetylated peptoids. In the case of the peptoid containing 4-aminoethylbenzoate, the resins were treated twice with acetyl chloride (50 eq.) and DIPEA (50 eq.) in THF 35 °C overnight.

### 3.3. General Procedure for TFA-Assisted Cleavage and Reverse-Phase HPLC

The peptoid-tethered resin, thoroughly washed with DCM, was suspended in a cleavage cocktail (Condition A: 50% TFA/3% TIS/5% H_2_O/42% DCM; Condition B: 92% TFA/3% TIS/5% H_2_O) for 1–3 h. After removing the cleavage solution by blowing with N_2_ gas, 50% aq. acetonitrile containing 0.05% TFA was added. The mixture was filtered through a 0.2 μm PTFE filter tip and freeze-dried. It was then redissolved in 50% aq. acetonitrile for HPLC and MALDI-TOF analyses. Reverse-phase HPLC experiments were conducted using a C18 reverse-phase column on a Shimadzu binary HPLC system equipped with a UV-visible detector set to 220 nm. The typical flow rate for analytical HPLC was 1 mL/min, employing a gradient elution of water/acetonitrile from 20% ACN/H_2_O to 80% ACN/H_2_O with 0.05% TFA over 40 min. Relative yields were determined by HPLC analysis based on the integration of each peak.

## 4. Conclusions

In summary, we systematically investigated the conformational and electronic effects of terminal side chains on the acid-catalyzed truncation of *N*-acylated peptoids. The experimental data conclusively demonstrated that both electronic and conformational effects of the terminal side chains are critical for truncation. The *n*→π* interaction plays a vital role in the proposed truncation mechanism. The electron-donating and steric effects of bulky branched side chains may facilitate the *n*→π*_Am_ interaction in the *trans*-amide conformation, thereby enhancing the formation of oxazolinium ion intermediate in the truncation mechanism. Conversely, electron-withdrawing *N*-aryl side chains significantly retard the truncation process. Along with our previous investigation into the electronic effects of terminal acyl groups [20], this study is crucial for understanding peptoid conformation and the unusual truncation behavior of *N*-acylated peptoids.

## Figures and Tables

**Table 1:** TFA-assisted truncation of peptoids with *N*-branched alkyl side chains ^a^.

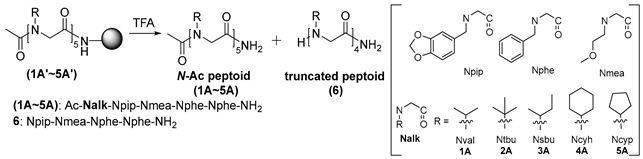		
Entry	Peptoid	Truncated Peptoid (6) (%) ^b^
1	**1A′**	100
2	**2A′**	100
3	**3A′**	100
4	**4A′**	100
5	**5A′**	93.4 (100) ^c^

^a^ Reaction conditions: 50% TFA/3% triisopropylsilane (TIS)/5% H_2_O/42% CH_2_Cl_2_, 1 h. ^b^ Relative yields of peptoids were determined by HPLC analysis. ^c^ Reaction time: 3 h.

**Table 2:** TFA-assisted truncation of peptoids with Ncyh unit.

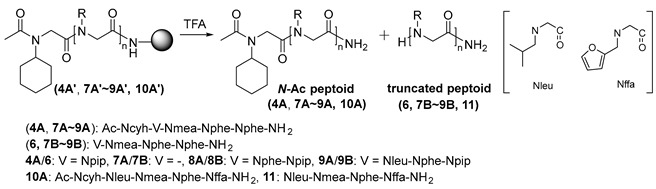					
Entry	Peptoid	Truncated Peptoid (%) ^a^			
		Cond. A ^b^		Cond. B ^c^	
		1 h	3 h	1 h	3 h
1	**7** **A′**	100	-	100	-
2	**4** **A′**	100	-	100	-
3	**8** **A′**	100	-	100	-
4	**9** **A′**	72.5	100	74.0	100
5	**10** **A′**	73.6	100	74.3	100

^a^ Relative yields of peptoids were determined by HPLC analysis. ^b^ Reaction conditions: 50% TFA/3% TIS/5% H_2_O/42% CH_2_Cl_2_. ^c^ Reaction conditions: 92% TFA/3% TIS/5% H_2_O.

**Table 3:** TFA-assisted truncation of peptoids with *N*-aryl side chains.

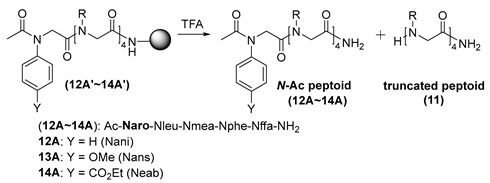					
Entry	Peptoid	p*K*a ^a^	Cond. ^b^	Truncated Peptoid (11) (%) ^c^	
				1 h	3 h
1	**12** **A′**	4.6	A	2.9	7.4
2			B	3.6	8.7
3	**13** **A′**	5.36	A	6.5	10.4
4			B	9.2	17.0
5	**14** **A′**	2.51	A	0.0	0.0
6			B	0.0	0.0

^a^ p*K*a values of the conjugate acids of the aryl amines. ^b^ Reaction conditions A: 50% TFA/3% TIS/5% H_2_O/42% CH_2_Cl_2_; B: 92% TFA/3% TIS/5% H_2_O. ^c^ Relative yields of peptoids were determined by HPLC analysis.

**Table 4:** TFA-assisted truncation of peptoids with Nans unit.

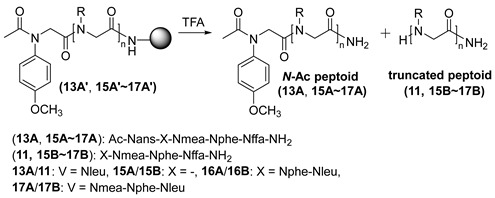					
Entry	Peptoid	Truncated Peptoid (%) ^a^			
		Cond. A ^b^		Cond. B ^c^	
		1 h	3 h	1 h	3 h
1	**15** **A′**	5.2	10.2	5.4	10.8
2	**13** **A′**	6.5	10.4	9.2	17.0
3	**16** **A′**	8.3	21.0	9.7	31.7
4	**17** **A′**	3.8	12.2	7.1	28.1

^a^ Relative yields of peptoids were determined by HPLC analysis. ^b^ Reaction conditions: 50% TFA/3% TIS/5% H_2_O/42% CH_2_Cl_2_. ^c^ Reaction conditions: 92% TFA/3% TIS/5% H_2_O.

**Table 5:** Comparison of *N*-branched and *N*-unbranched alkyl side chains on TFA-assisted truncation of peptoids.

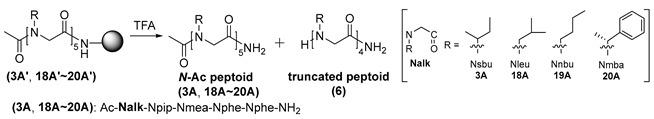					
Entry	Peptoid	Truncated Peptoid (6) (%) ^a^			
		Cond. A ^b^		Cond. B ^c^	
		1 h	3 h	1 h	3 h
1	**3** **A′**	100	-	100	-
2	**18** **A′**	65.5	84.0	69.8	85.8
3	**19** **A′**	44.0	76.7	69.4	78.5
4	**20** **A′**	70.0	86.7	86.9	100

^a^ Relative yields of peptoids were determined by HPLC analysis. ^b^ Reaction conditions: 50% TFA/3% TIS/5% H_2_O/42% CH_2_Cl_2_. ^c^ Reaction conditions: 92% TFA/3% TIS/5% H_2_O.

## Data Availability

The data are included in the article or the Supplementary Material. Additional data from this study are available upon request from the authors.

## Supplementary Materials

The following supporting information can be downloaded at https://www.mdpi.com/article/10.3390/ijms252111390/s1.
