# Supplementary material for: Systematic Investigation on Acid-Catalyzed Truncation of N-Acylated Peptoids
Source: Int J Mol Sci. 2024 Oct 23;25(21):11390. doi: 10.3390/ijms252111390 (PMC11547061; doi:10.3390/ijms252111390)
Supplement: Supplementary file 1 [file ijms-25-11390-s001.zip › ijms-3254264-supplementary.pdf]

Supplementary Materials

Systematic Investigation on Acid-Catalyzed Truncation of N-Acylated Peptoids

Ruiqi Piao and Yong-Uk Kwon*

Department of Chemistry and Nanoscience, Ewha Womans University, Seoul 03760, Korea
e-mail: yukwon@ewha.ac.kr

Contents

– Table S1. TFA-assisted truncation of peptoids with *N*-branched alkyl side chains 2

– Figures S1–S8. HPLC chromatograms and MALDI-TOF spectra of peptoids in Table S1 2–6

– Table S2. TFA-assisted truncation of peptoids with Ncyh unit 7

– Figures S9–S19. HPLC chromatograms and MALDI-TOF spectra of peptoids in Table S2 7–13

– Synthesis and acid-assisted truncation of peptoid (**4A**) using photo-cleavable ANP linker 14

– Scheme S1. Synthesis of peptoid (**4A**) using photo-cleavable ANP linker 14

– Figures S20–S21. HPLC chromatograms and MALDI-TOF spectra of peptoid **4A** 15

– Table S3. TFA-assisted truncation of peptoids with *N*-aryl side chains 16

– Figures S22–S29. HPLC chromatograms and MALDI-TOF spectra of peptoids in Table S3 16–20

– Table S4. TFA-assisted truncation of peptoids with Nans unit 21

– Figures S30–S38. HPLC chromatograms and MALDI-TOF spectra of peptoids in Table S4 21–27

– Table S5. Comparison of *N*-branched and *N*-unbranched alkyl side chains on TFA-assisted truncation of peptoids 28

– Figures S39–S47. HPLC chromatograms and MALDI-TOF spectra of peptoids in Table S5 28–33

Table S1. TFA-assisted truncation of peptoids with *N*-branched alkyl side chains [a]

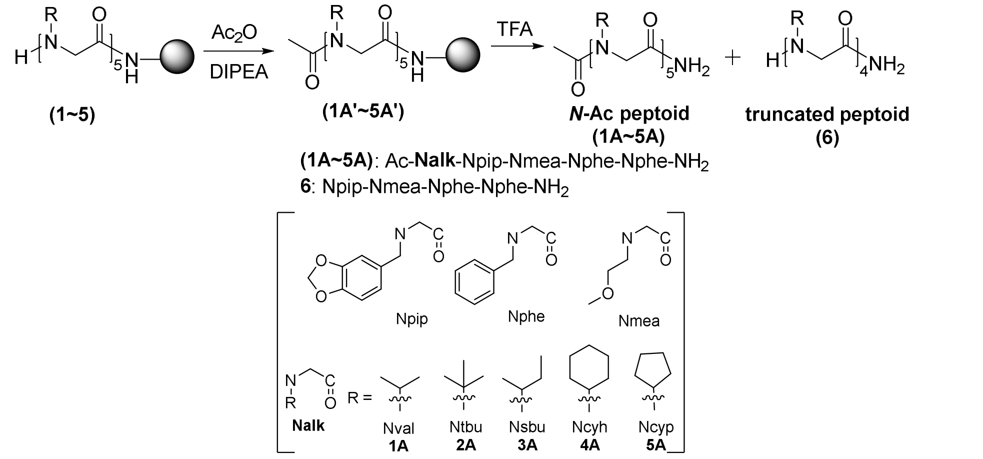

Entry	Peptoid	Truncated peptoid (**6**) (%) [b]
1	**1A′**	100
2	**2A′**	100
3	**3A′**	100
4	**4A′**	100
5	**5A′**	93.4 (100) [c]

[a] Reaction conditions: 50% TFA/3% triisopropylsilane (TIS)/5% H_2O/42% CH_2Cl_2, 1 h.
[b] Relative yields of peptoids were determined by HPLC analysis.
[c] Reaction time: 3 h.

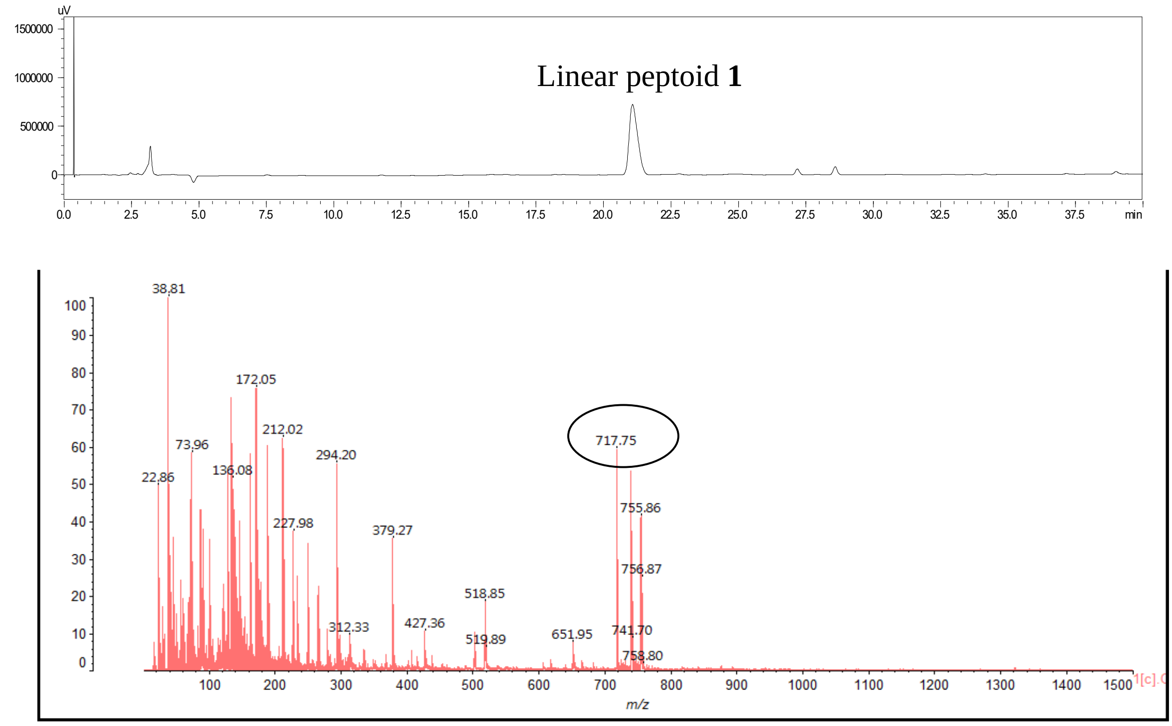

Figure S1. HPLC chromatogram and MALDI-TOF spectrum of linear peptoid **1**

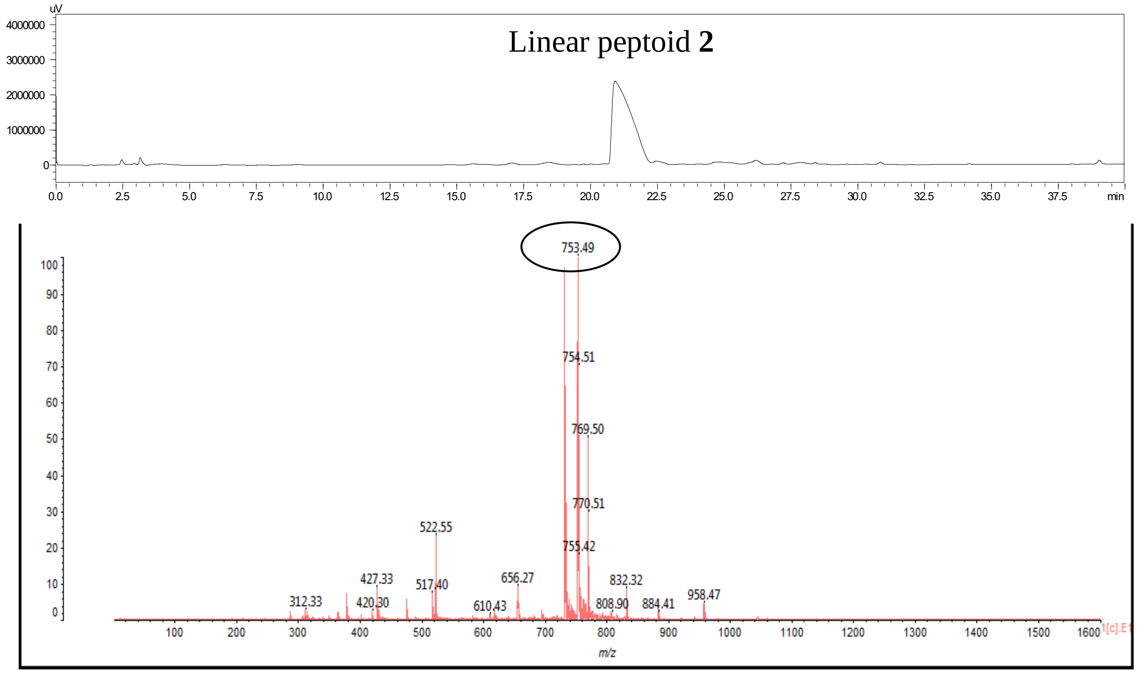

Figure S2. HPLC chromatogram and MALDI-TOF spectrum of linear peptoid **2**

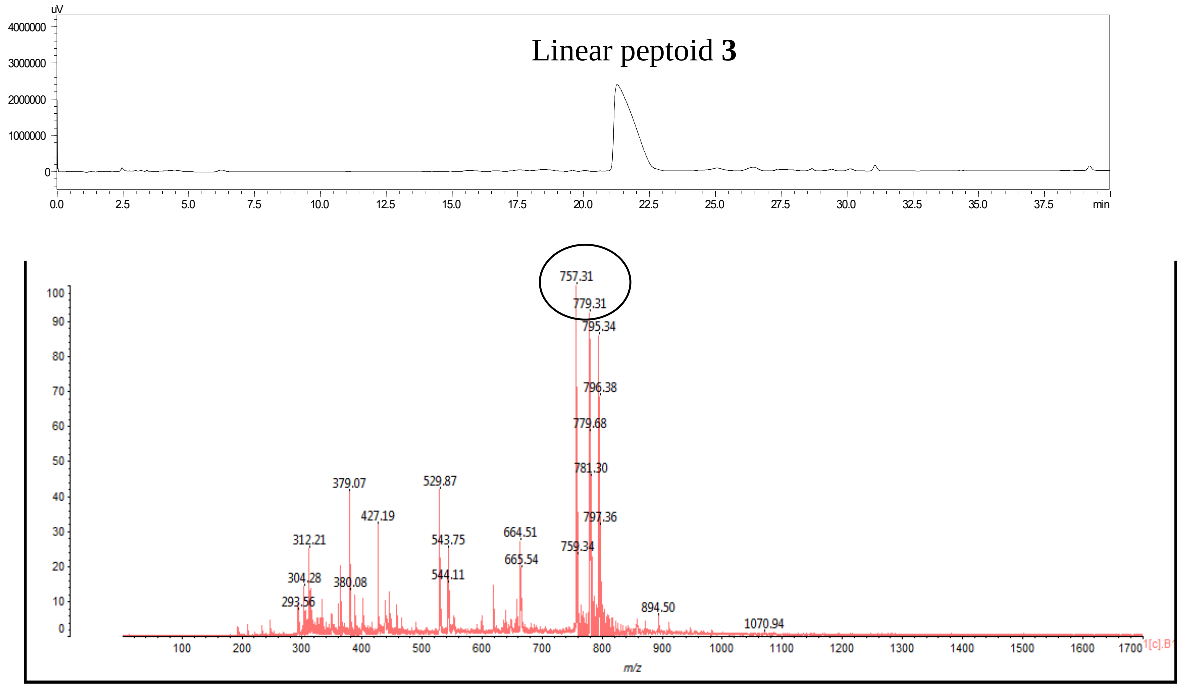

Figure S3. HPLC chromatogram and MALDI-TOF spectrum of linear peptoid **3**

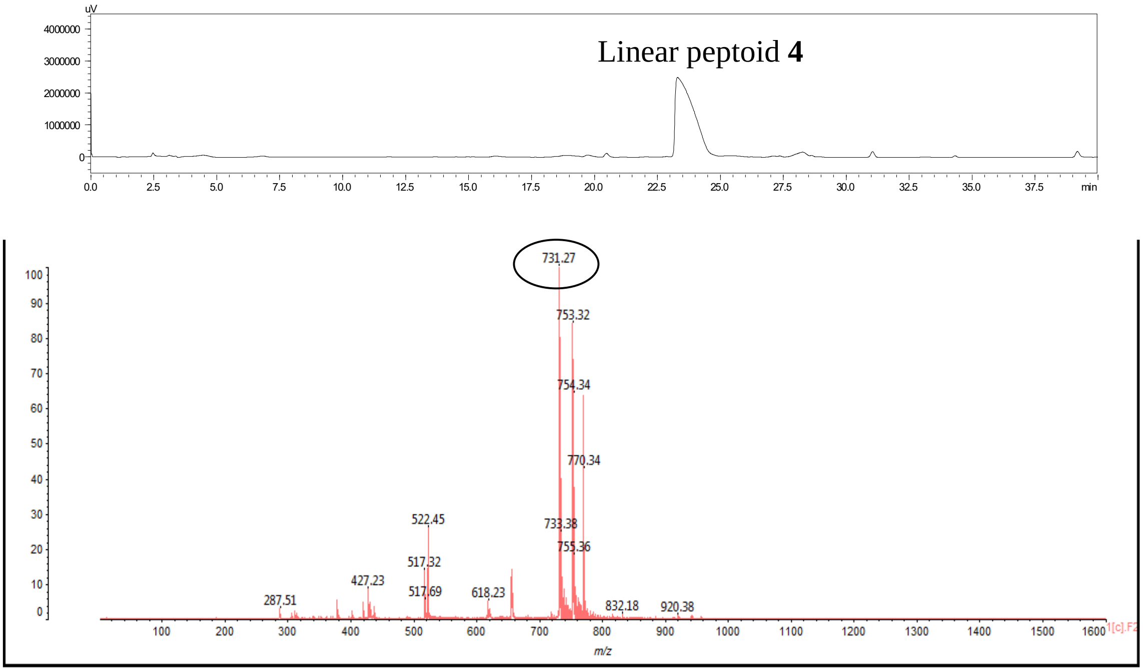

Figure S4. HPLC chromatogram and MALDI-TOF spectrum of linear peptoid **4**

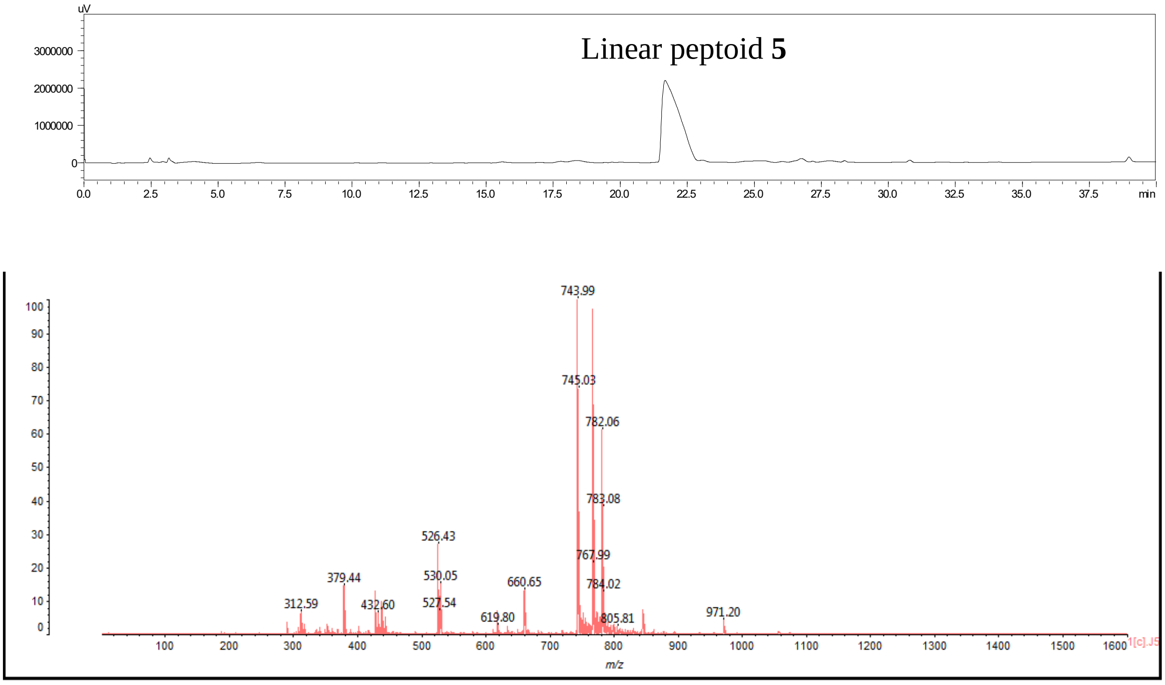

Figure S5. HPLC chromatogram and MALDI-TOF spectrum of linear peptoid **5**

1A’

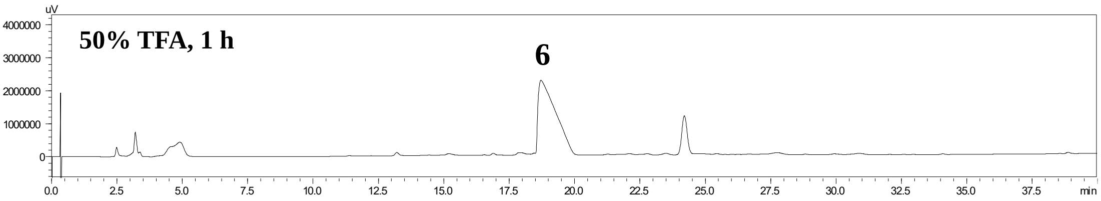

2A′

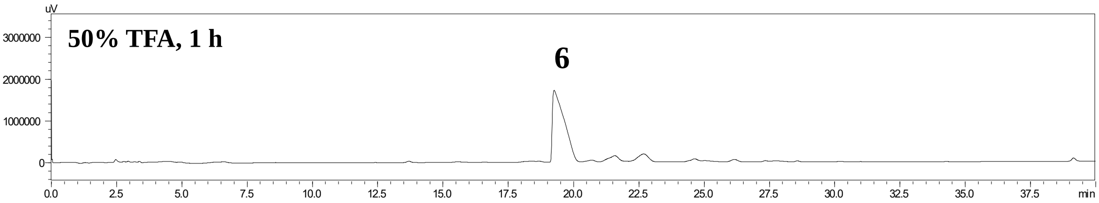

3A′

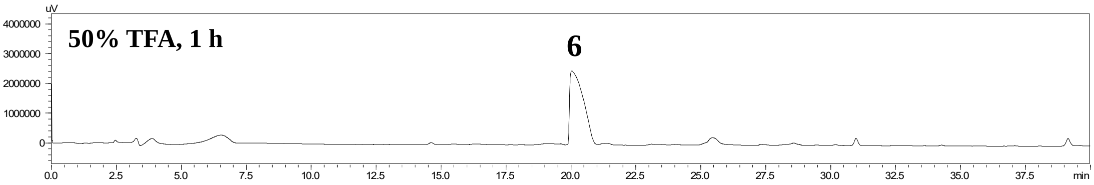

4A'

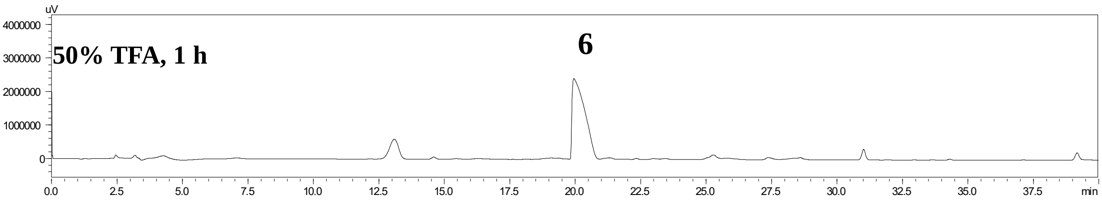

5A′

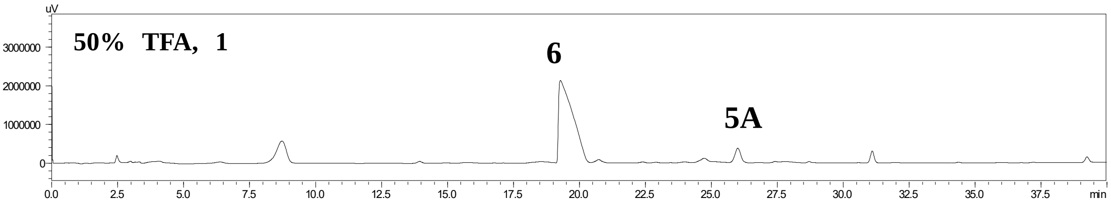

5A′

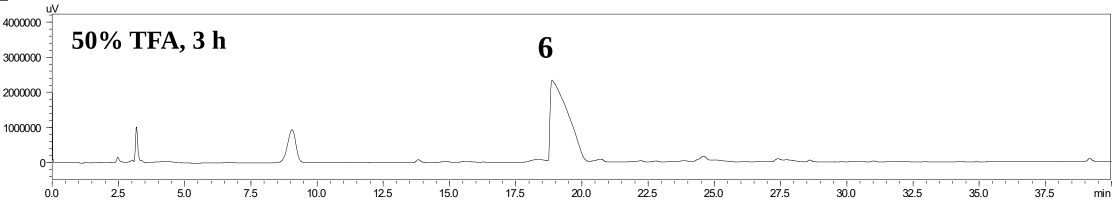

Figure S6. HPLC chromatograms after TFA-assisted truncation of peptoids **1A’–5A’**

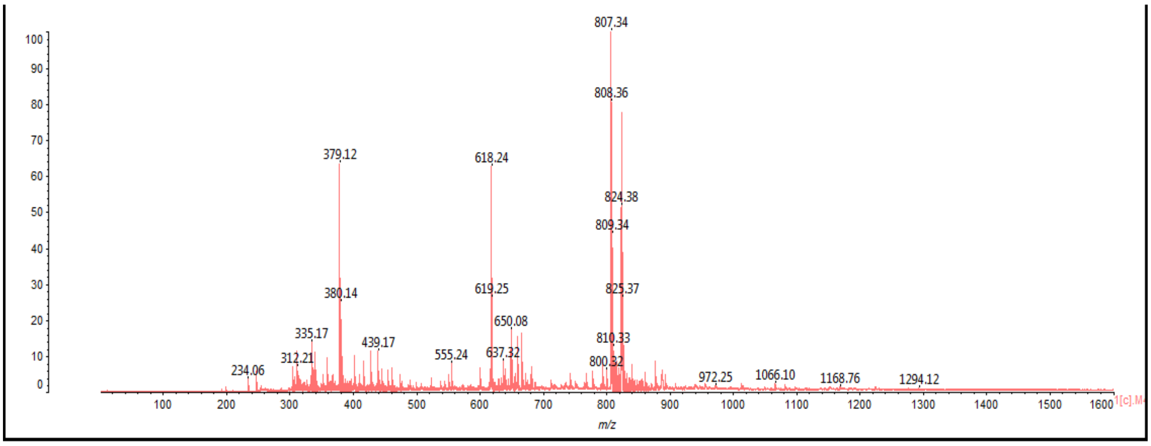

Figure S7. MALDI-TOF spectrum of linear peptoid **5A**

(a)

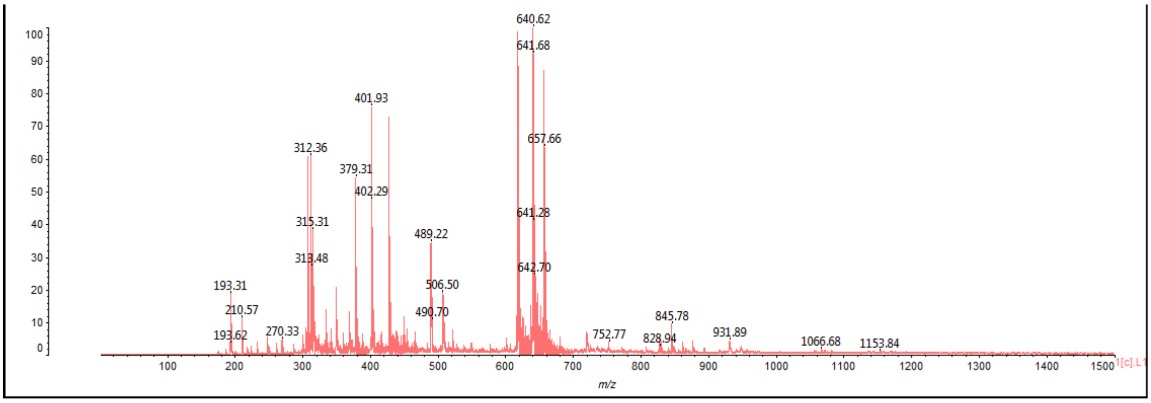

(b)

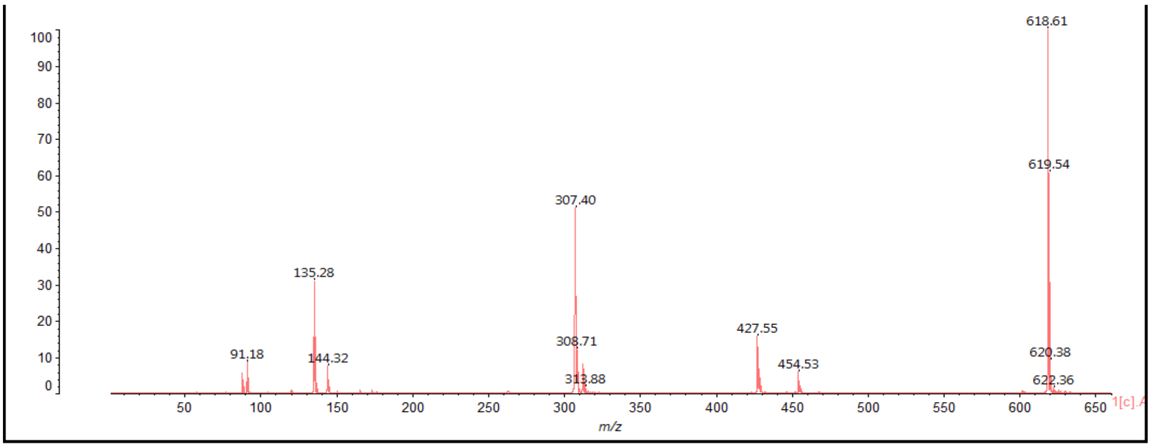

Figure S8. (a) MALDI-TOF and (b) MALDI-TOF/TOF spectra of truncated peptoid **6**

Table S2. TFA-assisted truncation of peptoids with Ncyh unit

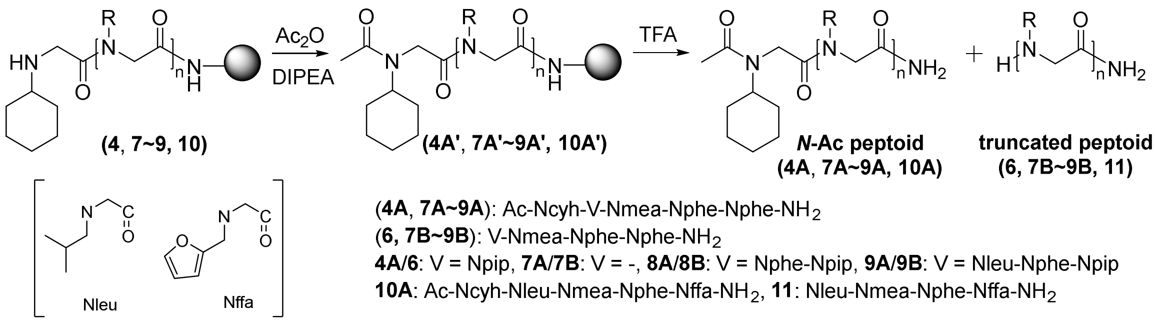

(**4A**, **7A~9A**): Ac-Ncyh-V-Nmea-Nphe-Nphe-NH_2
(**6, 7B~9B**): V-Nmea-Nphe-Nphe-NH_2
4A/6: V = Npip, **7A/7B**: V = -, **8A/8B**: V = Nphe-Npip, **9A/9B**: V = Nleu-Nphe-Npip
10A: Ac-Ncyh-Nleu-Nmea-Nphe-Nffa-NH_2, **11**: Nleu-Nmea-Nphe-Nffa-NH_2

Entry	Peptoid	Truncated peptoid (%) [a]			
		Cond. A [b]		Cond. B [c]	
		1 h	3 h	1 h	3 h
1	**7A′**	100	-	100	-
2	**4A′**	100	-	100	-
3	**8A′**	100	-	100	-
4	**9A′**	72.5	100	74.0	100
5	**10A′**	73.6	100	74.3	100

[a] Relative yields of peptoids were determined by HPLC analysis.
[b] Reaction conditions: 50% TFA/3% TIS/5% H_2O/42% CH_2Cl_2.
[c] Reaction conditions: 92% TFA/3% TIS/5% H_2O.

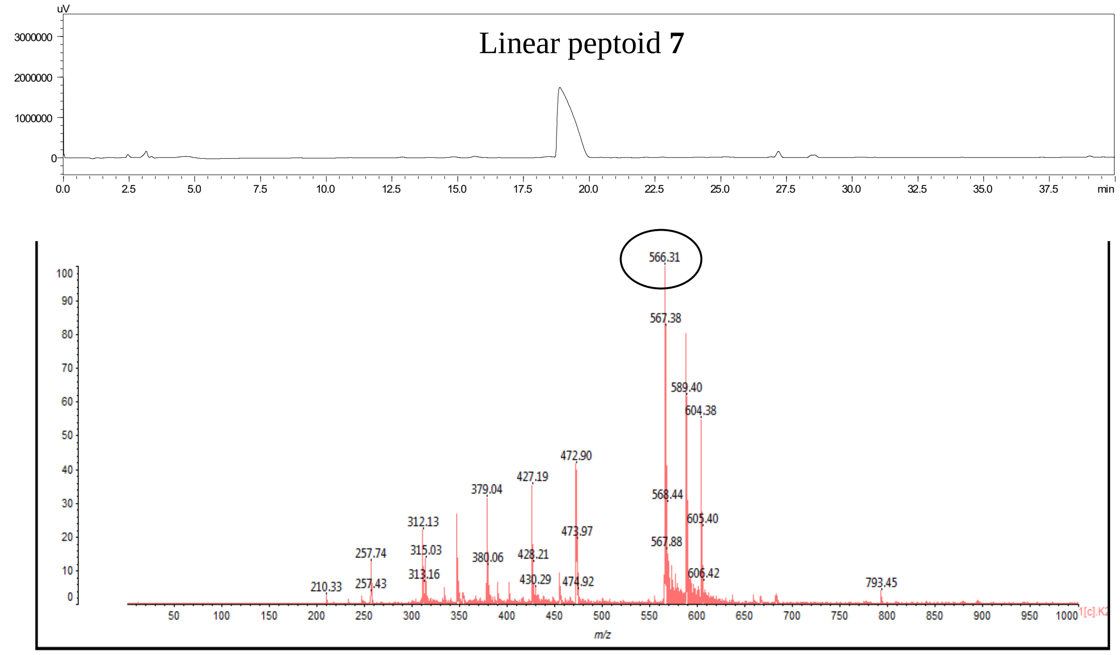

Figure S9. HPLC chromatogram and MALDI-TOF spectrum of linear peptoid **7**

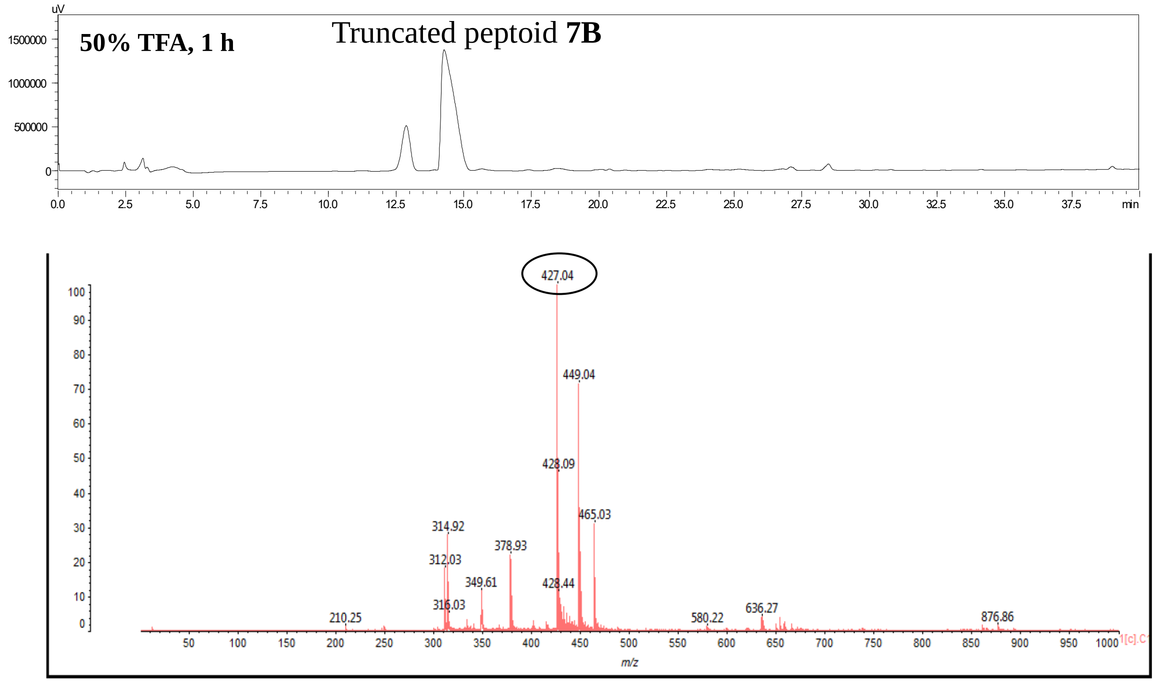

Figure S10. HPLC chromatogram and MALDI-TOF spectrum of truncated peptoid **7B**

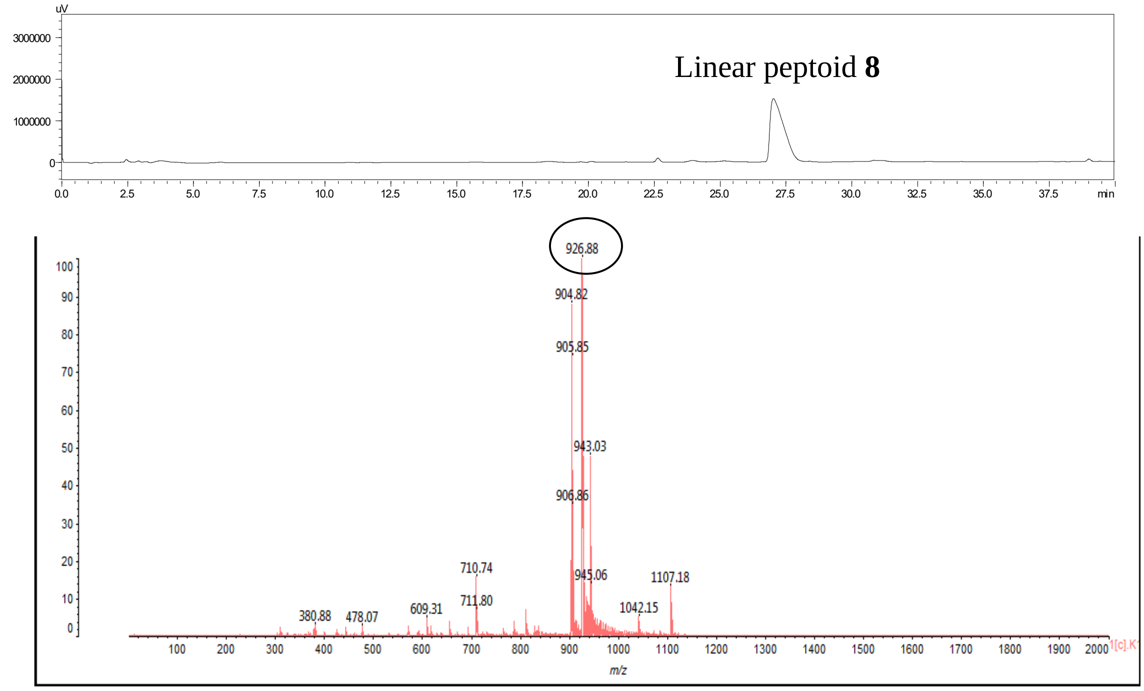

Figure S11. HPLC chromatogram and MALDI-TOF spectrum of linear peptoid **8**

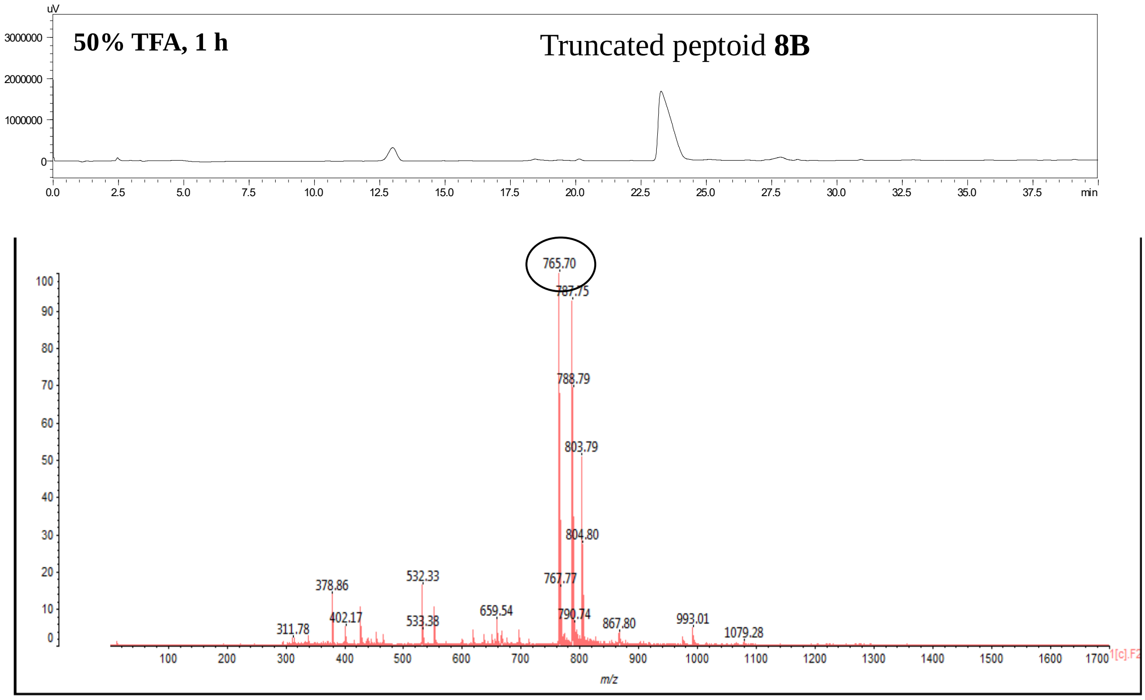

Figure S12. HPLC chromatogram and MALDI-TOF spectrum of truncated peptoid **8B**

Linear peptoid **9**

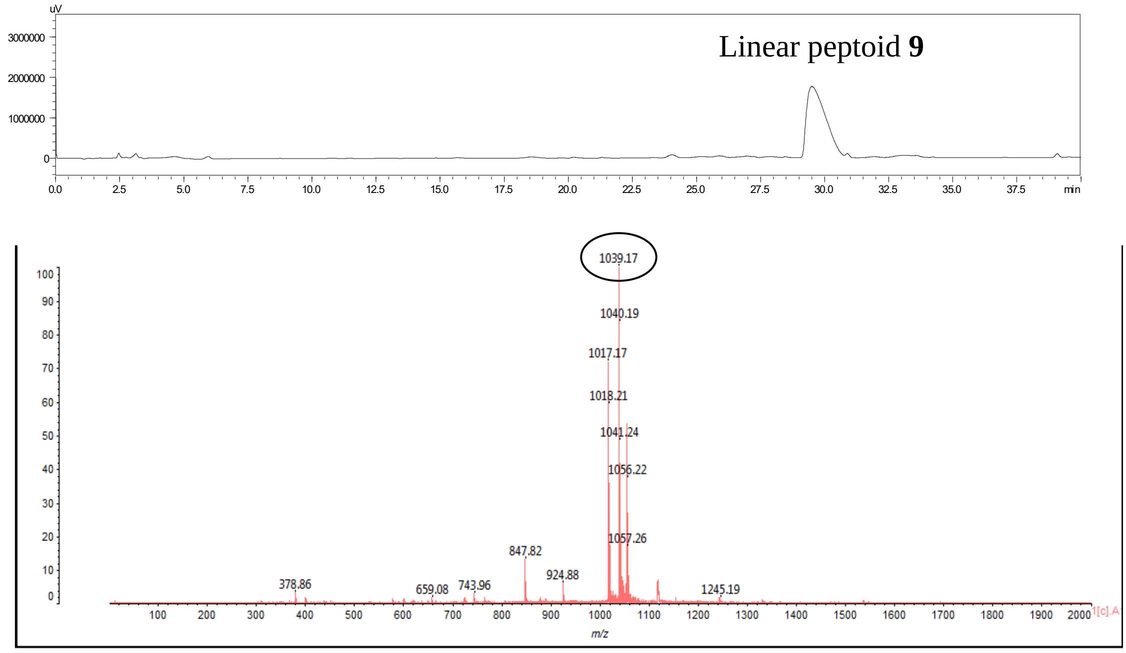

Figure S13. HPLC chromatogram and MALDI-TOF spectrum of linear peptoid **9**

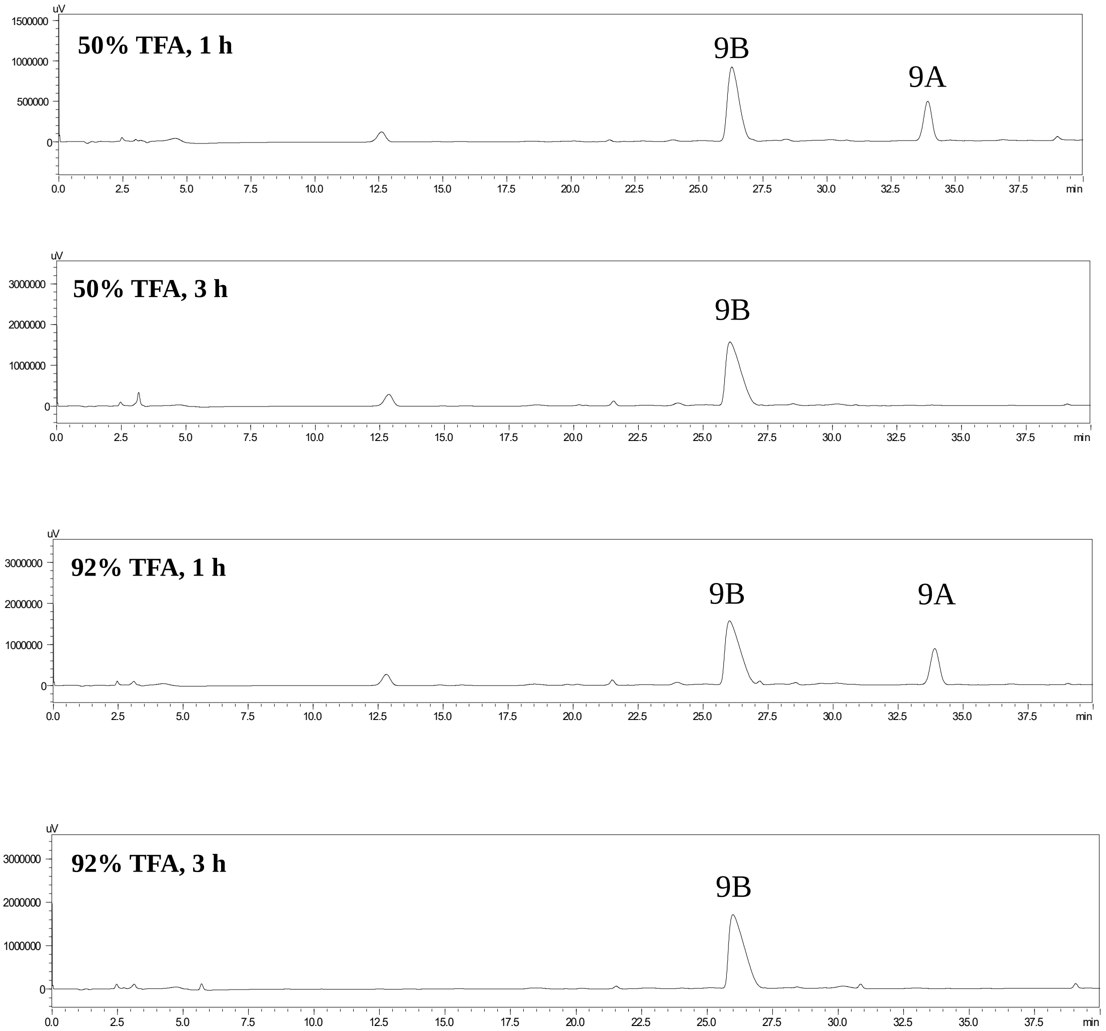

Figure S14. HPLC chromatograms after TFA-assisted truncation of peptoid **9A’**

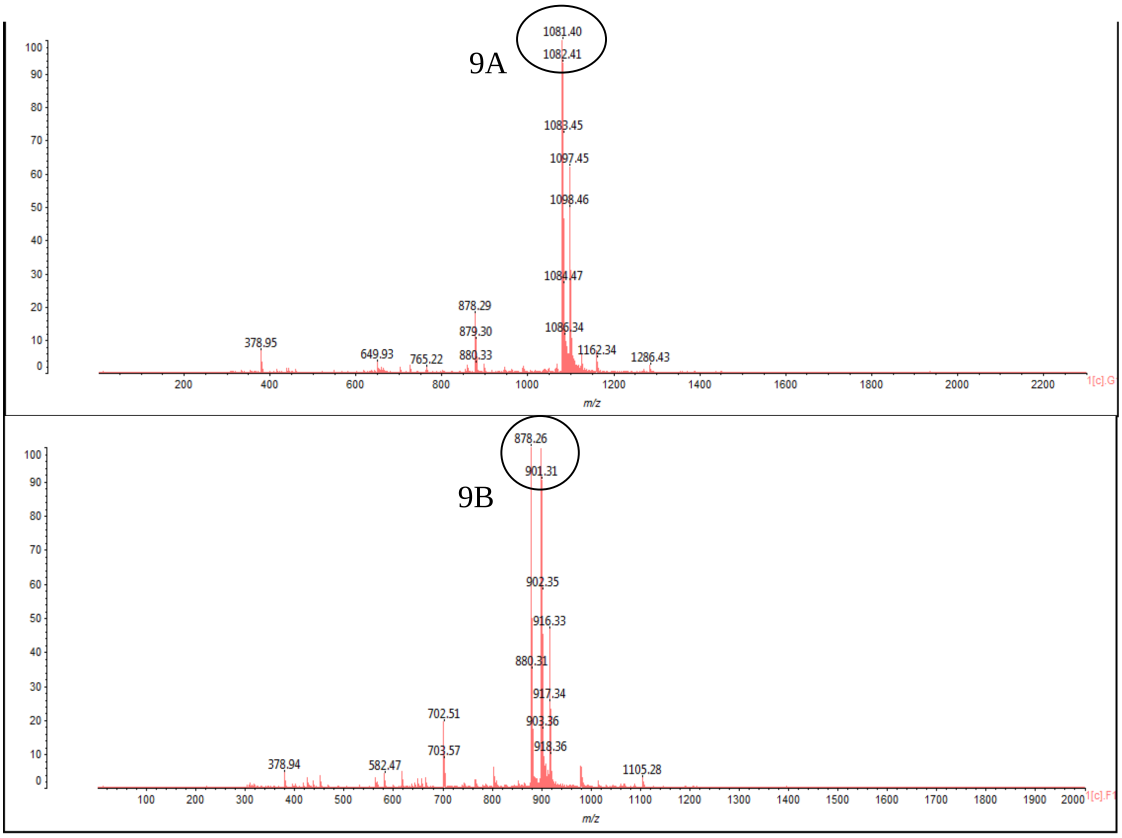

Figure S15. MALDI-TOF spectra of peptoids **9A** and **9B**

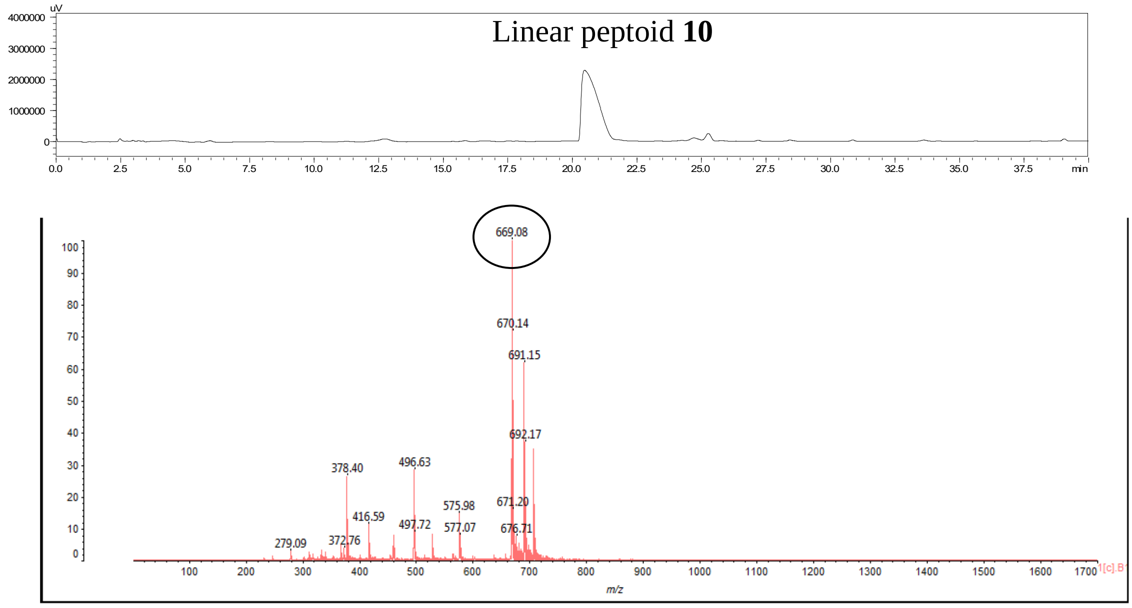

Figure S16. HPLC chromatogram and MALDI-TOF spectrum of linear peptoid **10**

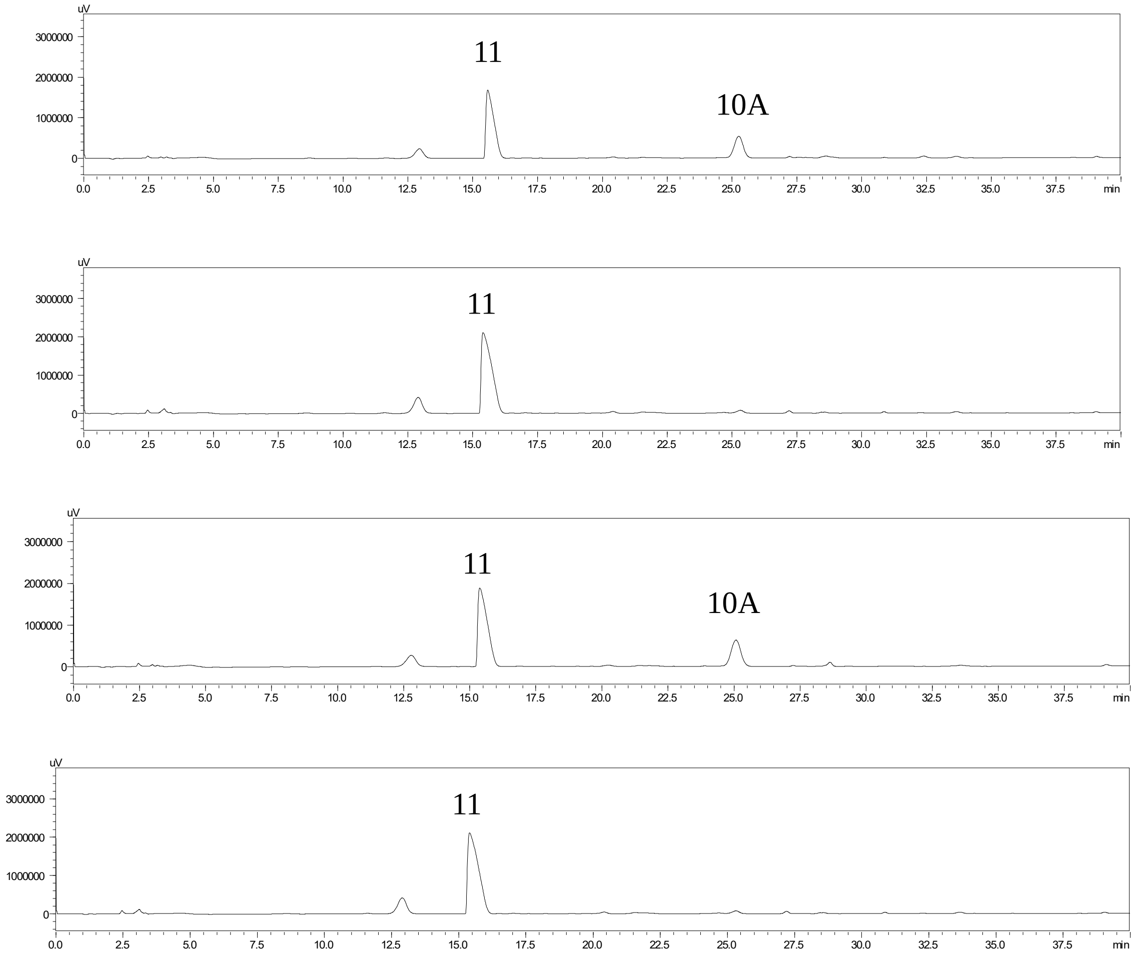

Figure S17. HPLC chromatograms after TFA-assisted truncation of peptoid **10A’**

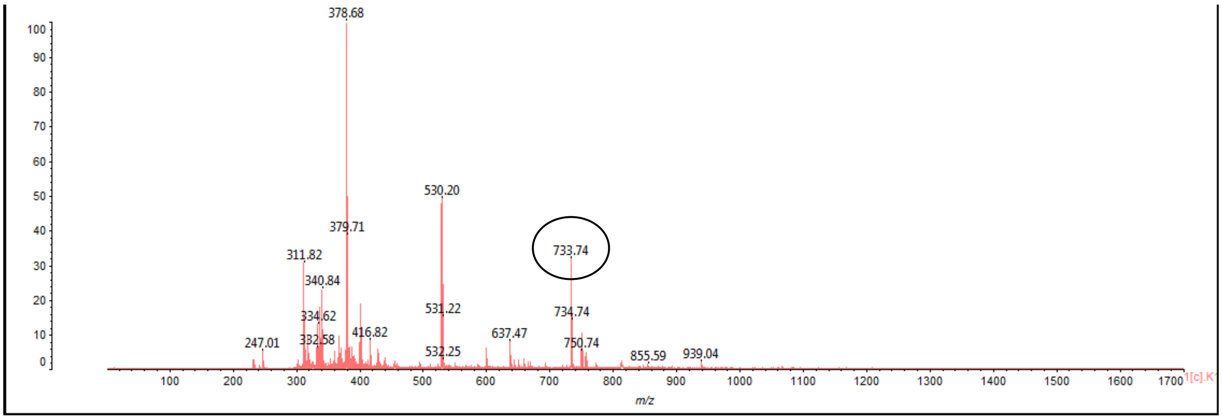

Figure S18. MALDI-TOF spectrum of peptoid **10A**

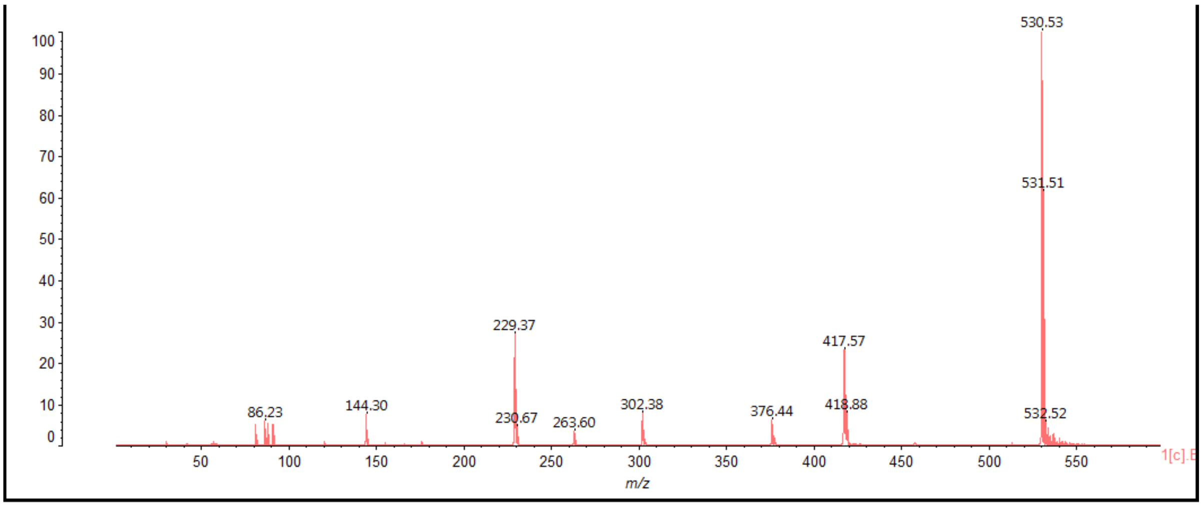

Figure S19. MALDI-TOF/TOF spectrum of truncated peptoid **11**

Synthesis and acid-assisted truncation of peptoid (**4A**) using photo-cleavable ANP linker

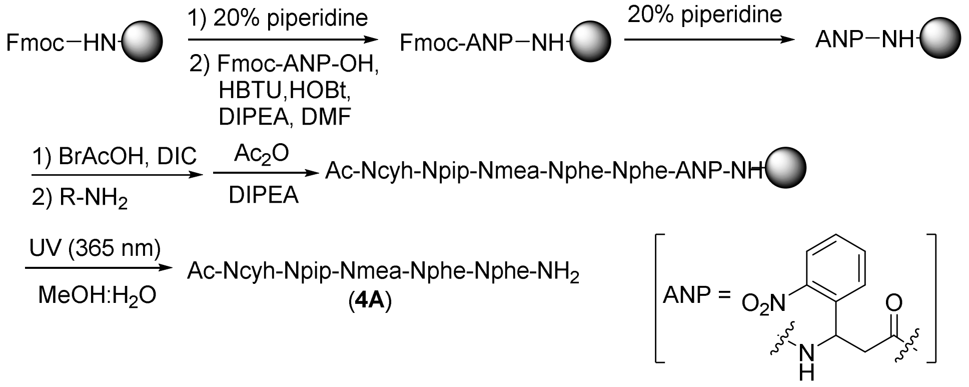

Scheme S1. Synthesis of peptoid (**4A**) using ANP linker

Synthesis: TentaGel MB RAM resins were swelled in DMF at 25 °C for 1 h. Then DMF was drained, and the beads were incubated 20% piperidine in DMF for 1 h and washed thoroughly with DMF (8×3 ml). Then, the beads were treated with Fmoc-ANP-OH (5eq.), HBTU (5eq.), HOBt (5eq.) and DIPEA (10eq.) in 2 ml DMF in shaker at room temperature overnight. The desired peptoid sequence was constructed by the conventional submonomer strategy. After acetylation using Ac_2O, the peptoid was photo-cleaved by UV (365 nm) irradiation of the resin in MeOH/H_2O (1/1) for 3 h.

Acid-assisted truncation: condition A: 92% TFA/3% TIS/5% H_2O, 1 h; condition B: HCl (12 N)/MeOH (1/1), 1 h.

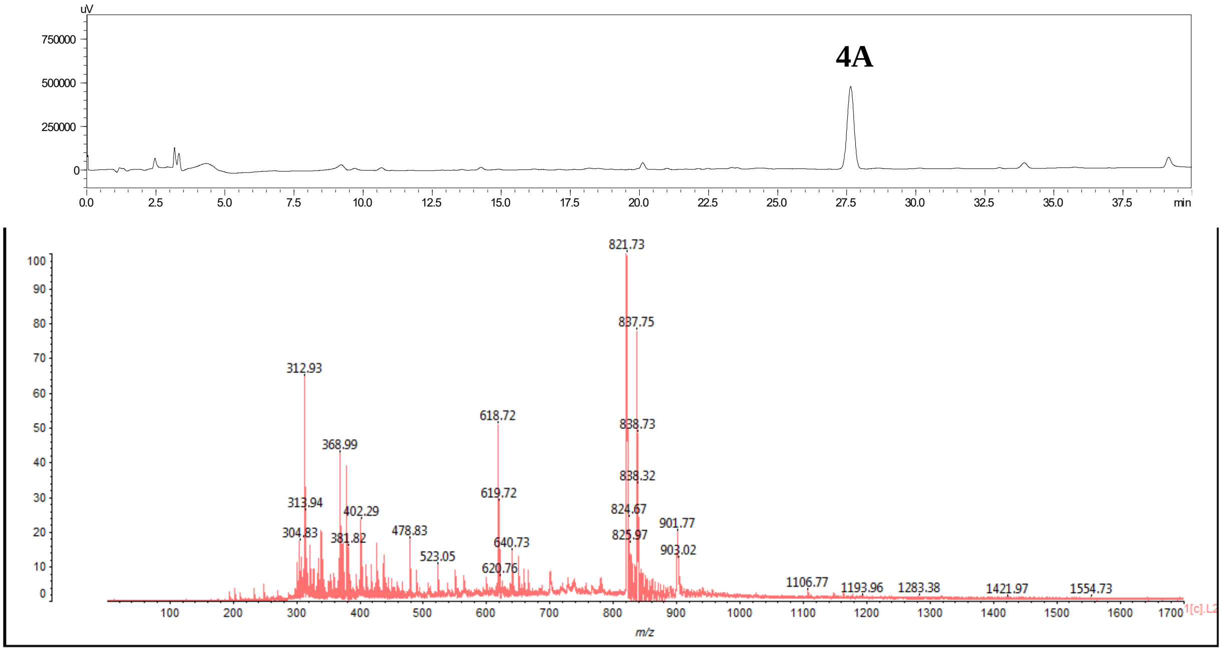

Figure S20. HPLC chromatogram and MALDI-TOF spectrum of acetylated peptoid **4A** after photo-cleavage

(a) 92% TFA, 1 h

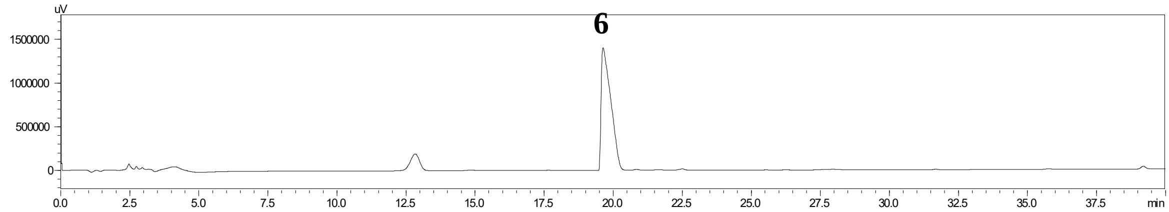

(b) HCl (12 N)/MeOH (1/1), 1 h

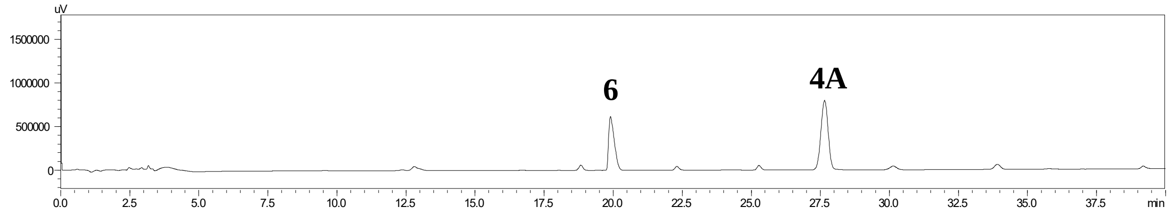

Figure S21. HPLC chromatograms after the acid-assisted truncation of acetylated peptoid **4A** in solution-phase

Table S3. TFA-assisted truncation of peptoids with *N*-aryl side chains

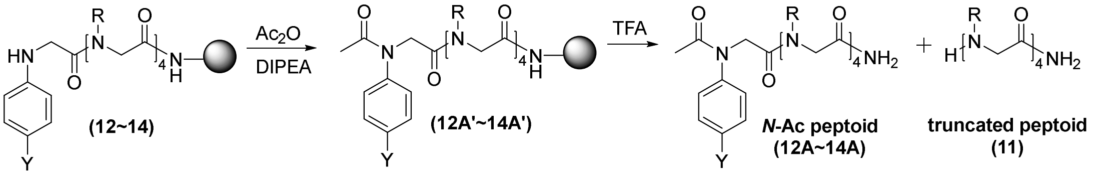

(**12A~14A**): Ac-**Naro**-Nleu-Nmea-Nphe-Nffa-NH_2
12A: Y = H (Nani)
13A: Y = OMe (Nans)
14A: Y = CO_2Et (Neab)

Entry	Peptoid	p*Ka* [a]	Cond. [b]	Truncated peptoid (**11**) (%) [c]	
				1 h	3 h
1	**12A′**	4.6	A	2.9	7.4
2			B	3.6	8.7
3	**13A′**	5.36	A	6.5	10.4
4			B	9.2	17.0
5	**14A′**	2.51	A	0.0	0.0
6			B	0.0	0.0

[a] p*Ka* values of the conjugate acids of the aryl amines.
[b] Reaction conditions A: 50% TFA/3% TIS/5% H_2O/42% CH_2Cl_2, B: 92% TFA/3% TIS/5% H_2O.
[c] Relative yields of peptoids were determined by HPLC analysis.

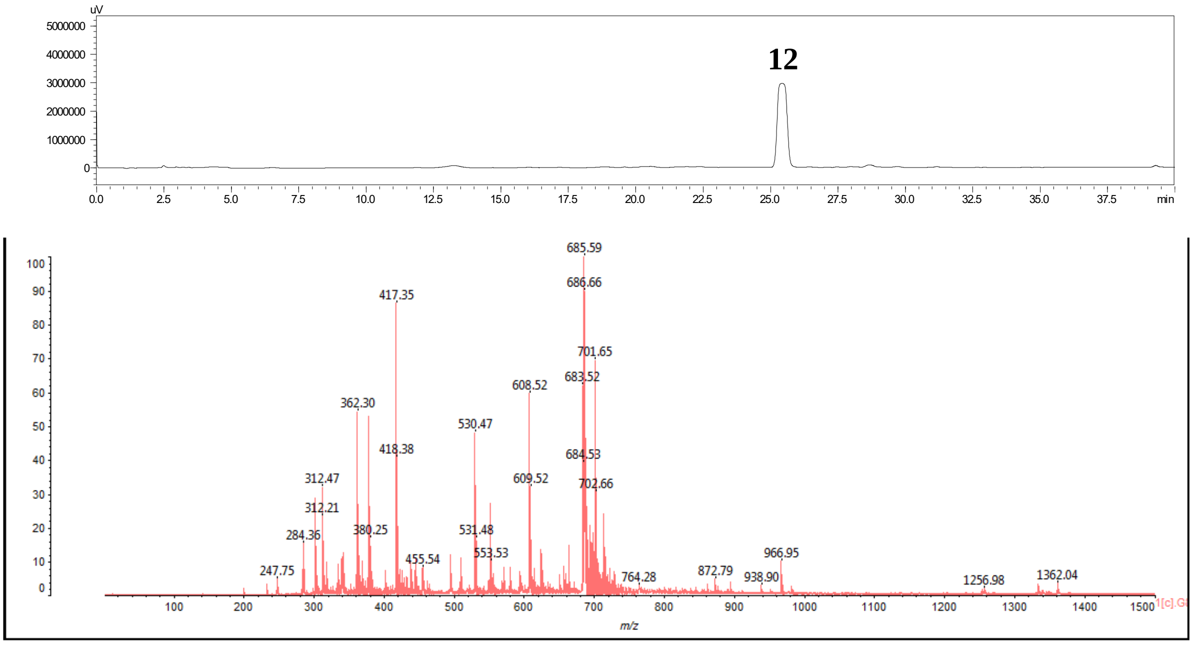

Figure S22. HPLC chromatogram and MALDI-TOF spectrum of linear peptoid **12**

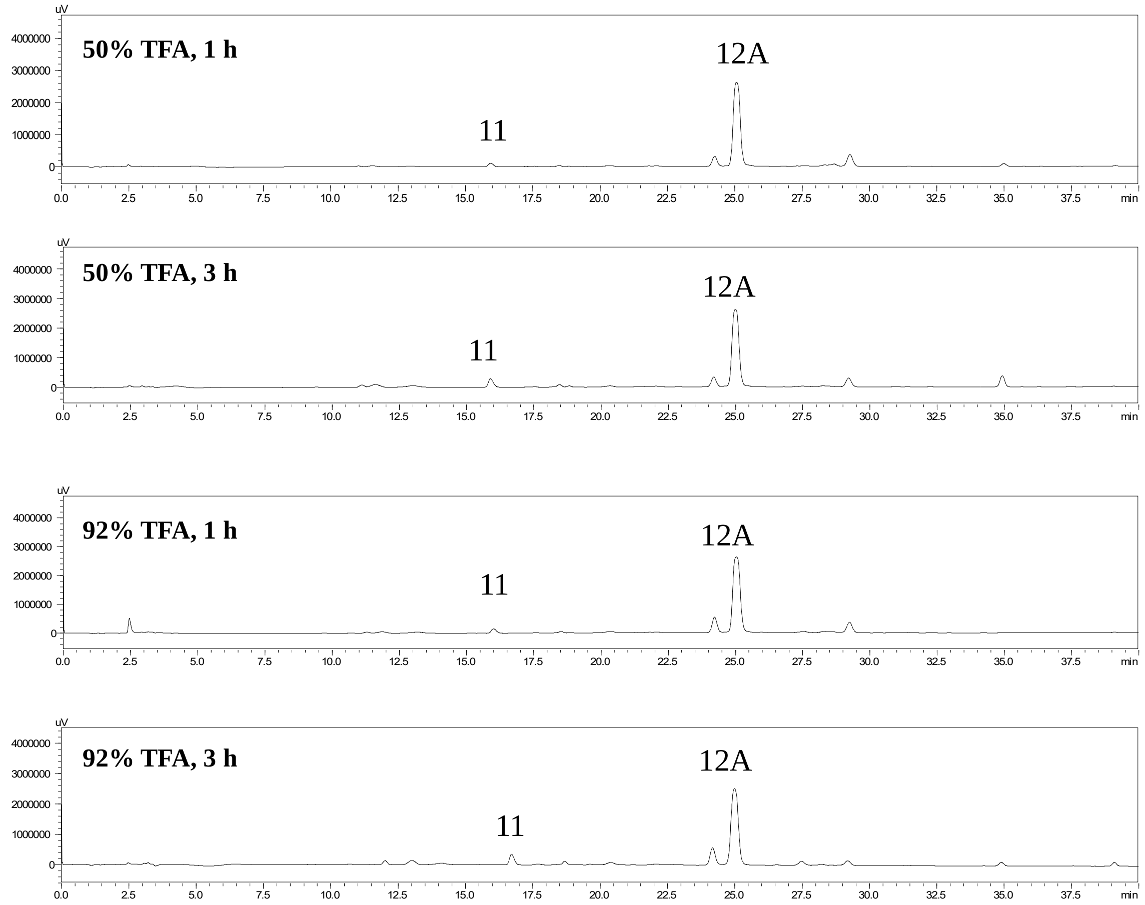

Figure S23. HPLC chromatograms after TFA-assisted truncation of peptoid **12A’**

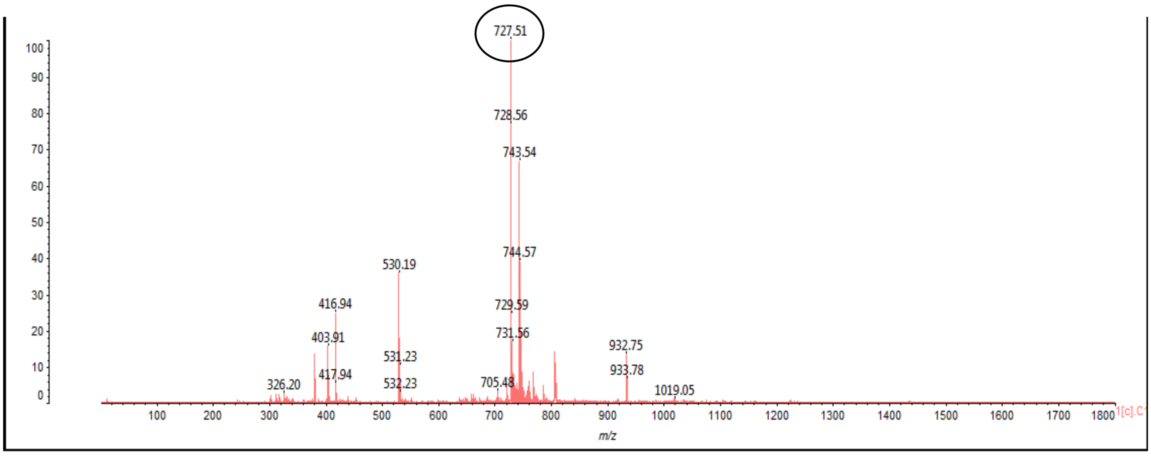

Figure S24. MALDI-TOF spectrum of peptoid **12A**

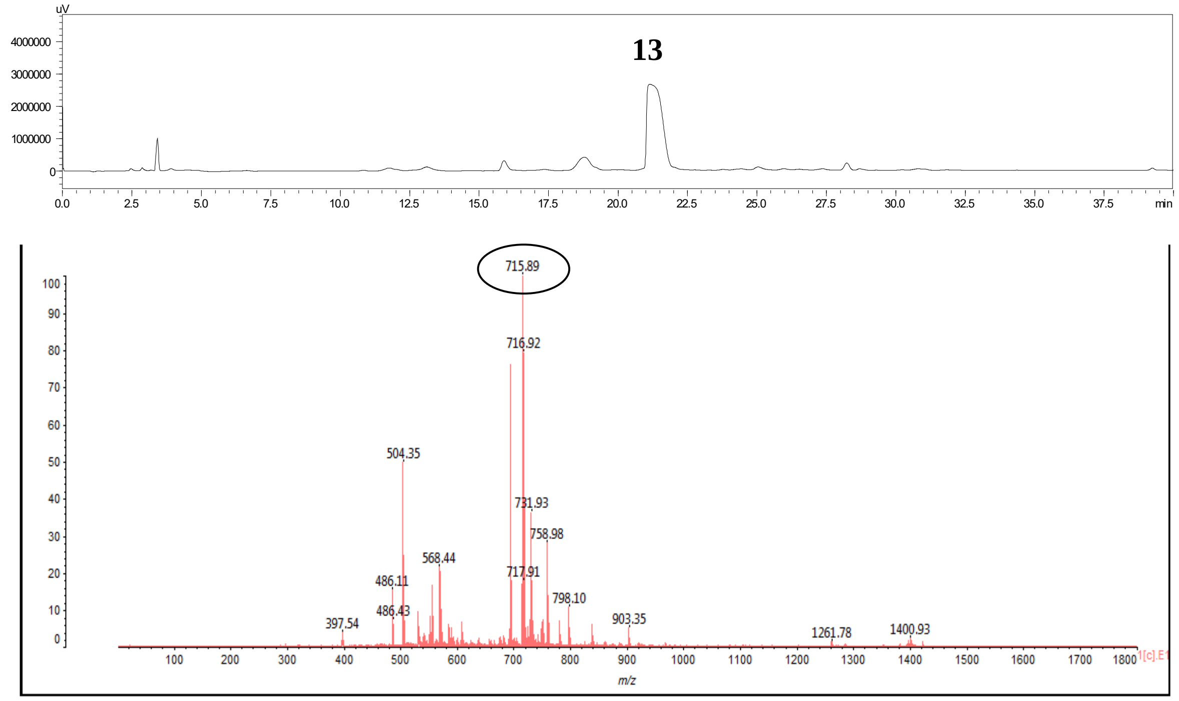

Figure S25. HPLC chromatogram and MALDI-TOF spectrum of linear peptoid **13**

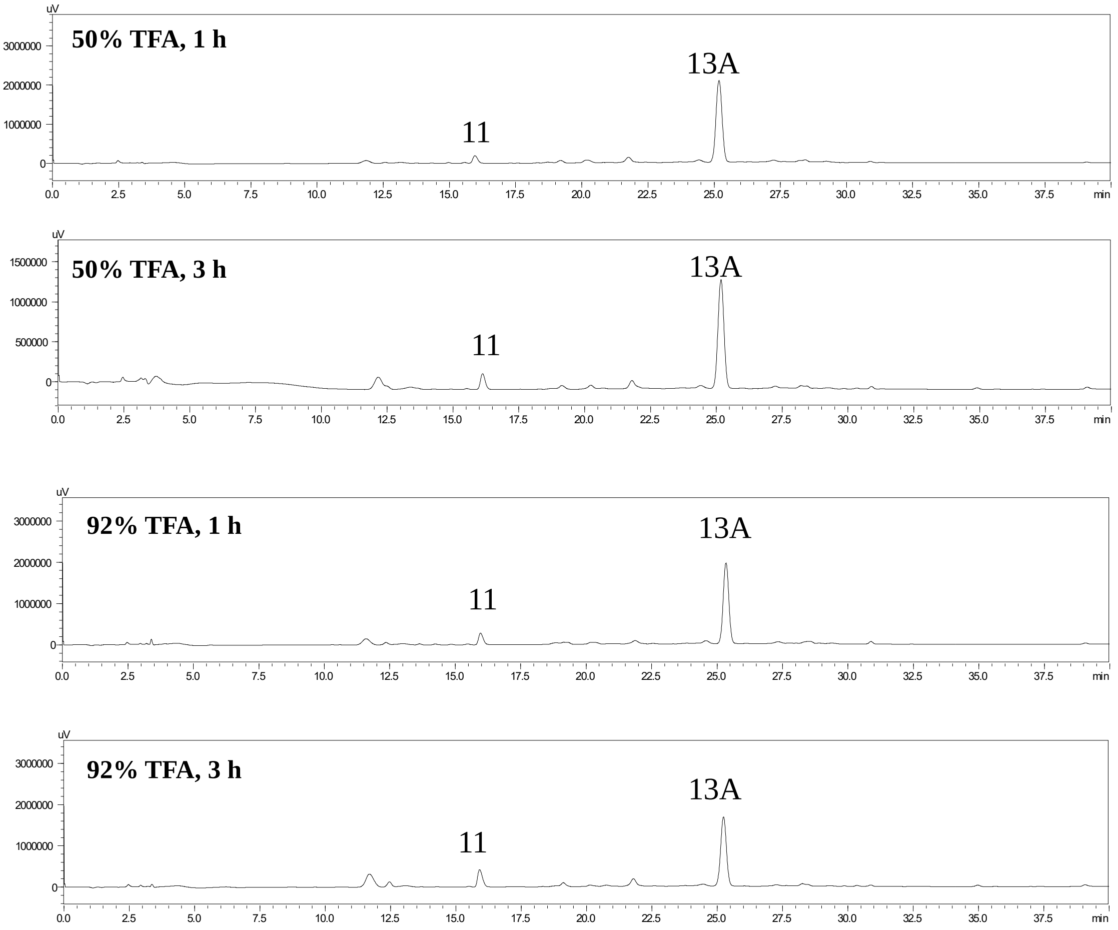

Figure S26. HPLC chromatograms after TFA-assisted truncation of peptoid **13A'**

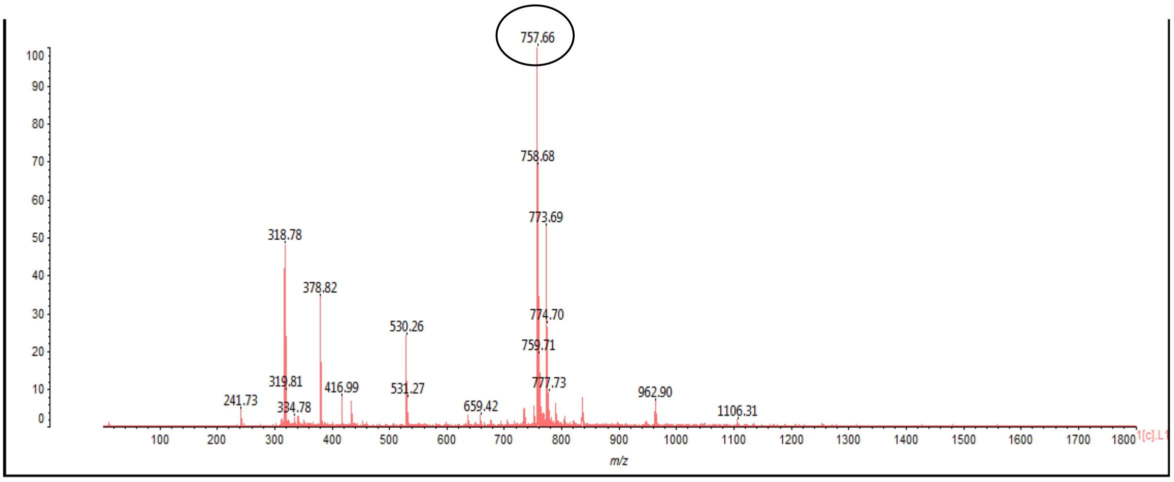

Figure S27. MALDI-TOF spectrum of peptoid **13A**

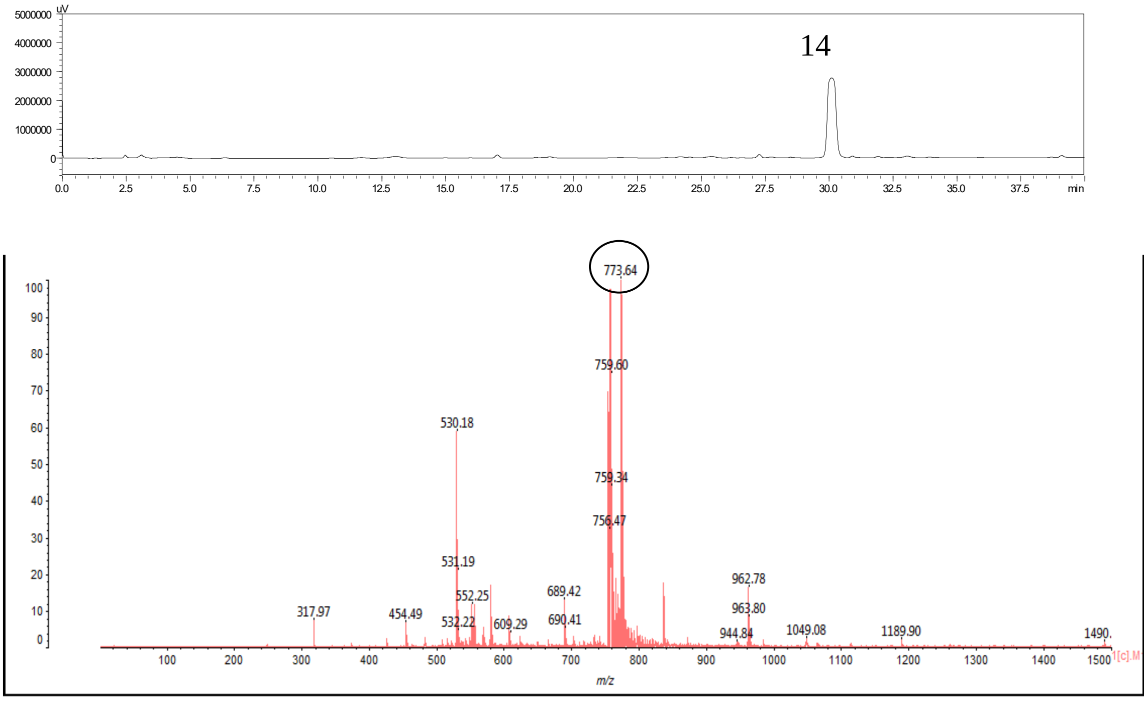

Figure S28. HPLC chromatogram and MALDI-TOF spectrum of linear peptoid **14**

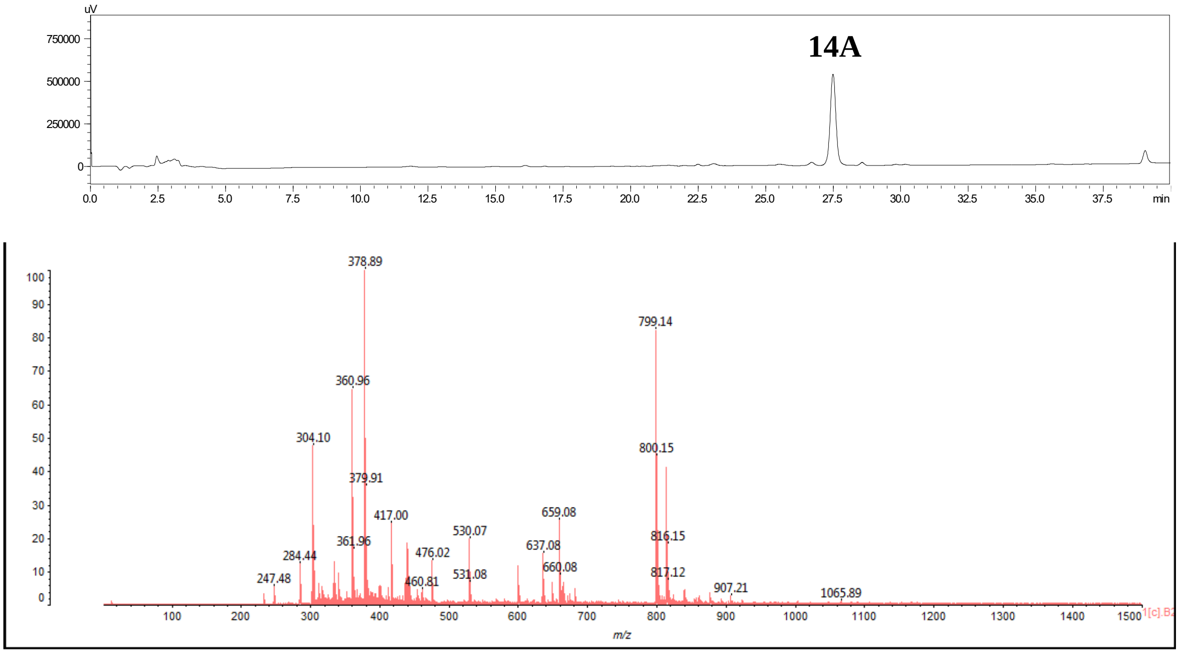

Figure S29. HPLC chromatogram and MALDI-TOF spectrum of peptoid **14A**

Table S4. TFA-assisted truncation of peptoids with Nans unit

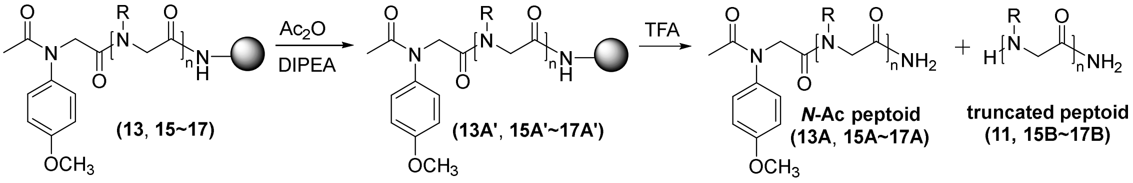

(**13A**, **15A~17A**): Ac-Nans-X-Nmea-Nphe-Nffa-NH$_2$
(**11, 15B~17B**): X-Nmea-Nphe-Nffa-NH$_2$
13A/11: V = Nleu, **15A/15B**: X = -, **16A/16B**: X = Nphe-Nleu,
17A/17B: V = Nmea-Nphe-Nleu

Entry	Peptoid	Truncated peptoid (%) [a]			
		Cond. A [b]		Cond. B [c]	
		1 h	3 h	1 h	3 h
1	**15A′**	5.2	10.2	5.4	10.8
2	**13A′**	6.5	10.4	9.2	17.0
3	**16A′**	8.3	21.0	9.7	31.7
4	**17A′**	3.8	12.2	7.1	28.1

[a] Relative yields of peptoids were determined by HPLC analysis.
[b] Reaction conditions: 50% TFA/3% TIS/5% H_2O/42% CH_2Cl_2.
[c] Reaction conditions: 92% TFA/3% TIS/5% H_2O.

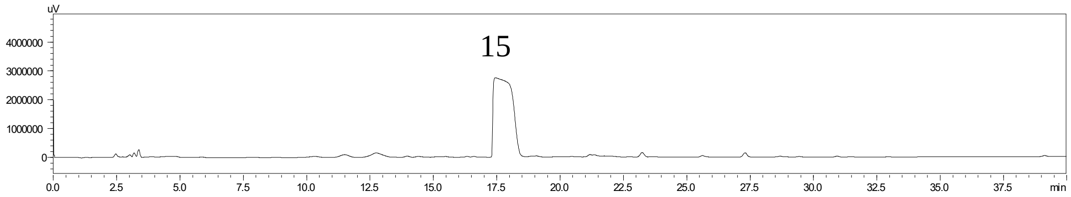

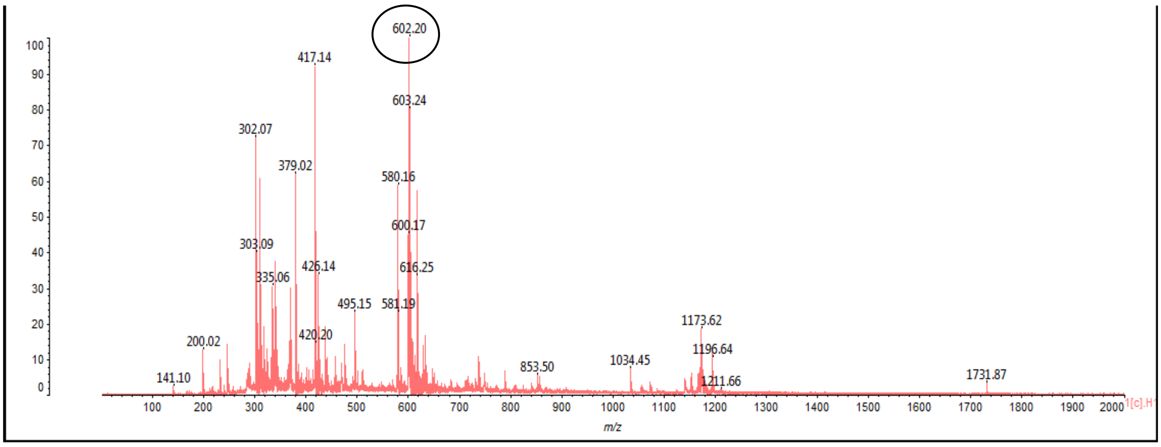

Figure S30. HPLC chromatogram and MALDI-TOF spectrum of peptoid **15**

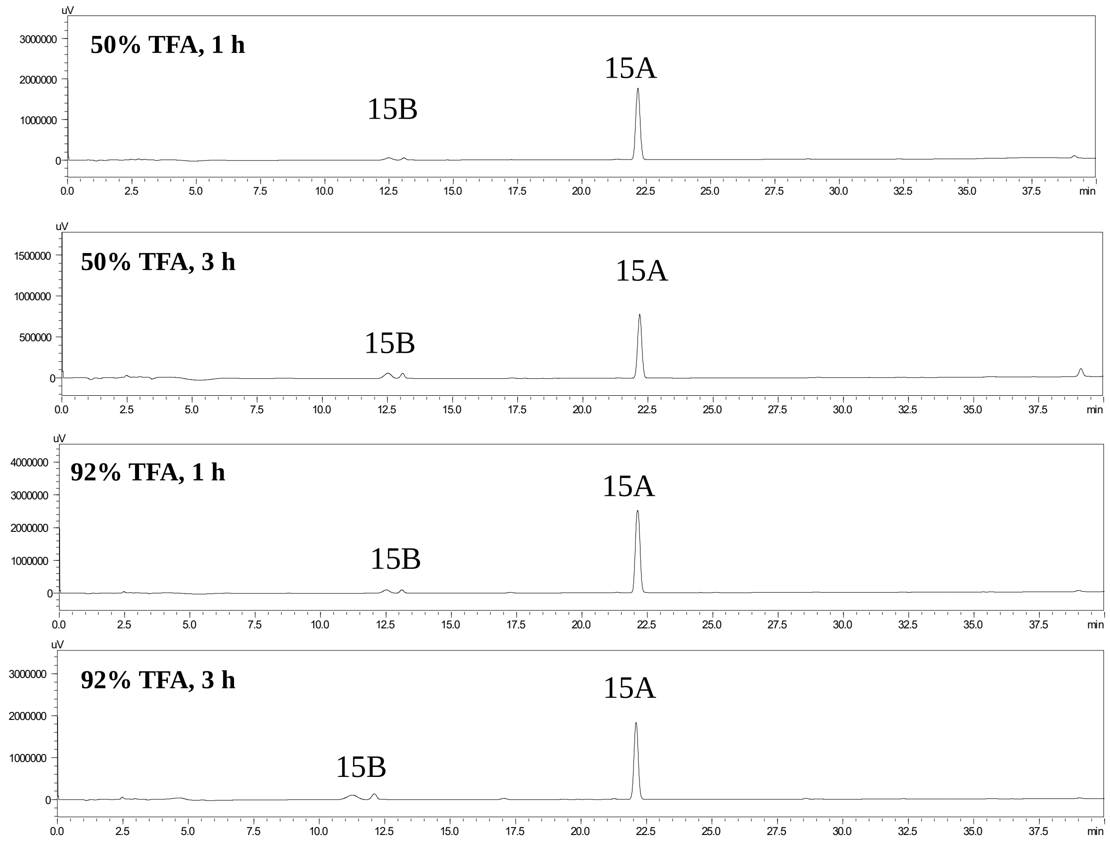

Figure S31. HPLC chromatograms after TFA-assisted truncation of peptoid **15A’**

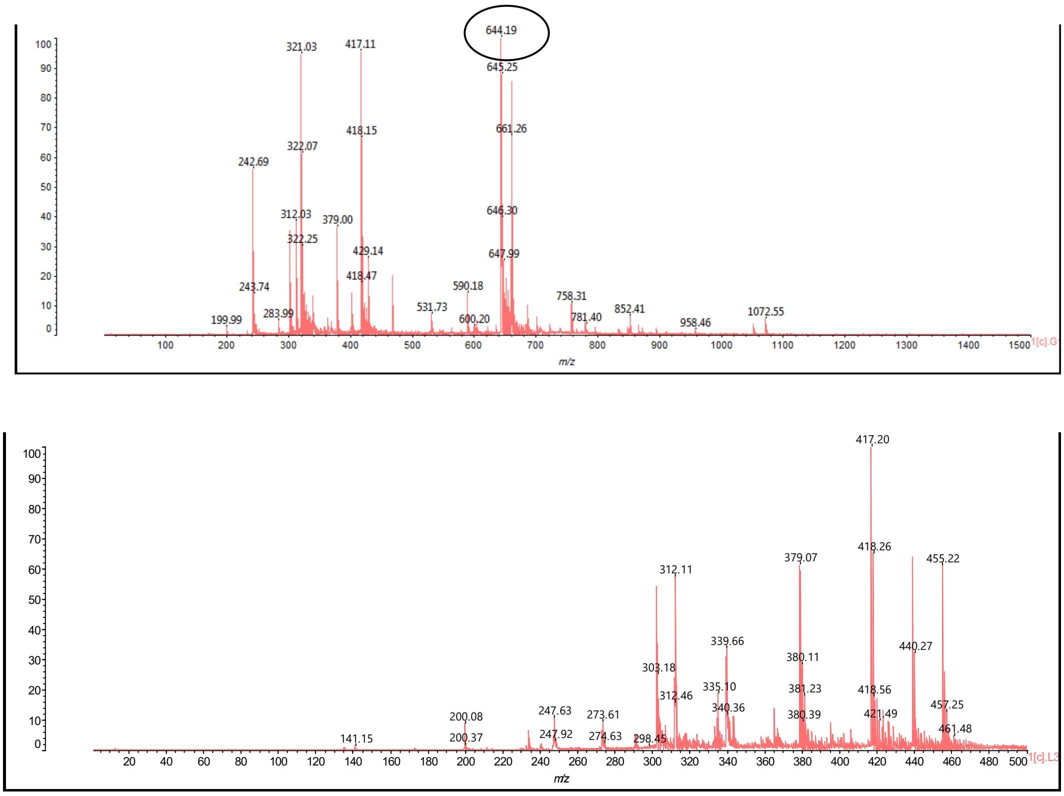

Figure S32. MALDI-TOF spectra of peptoids **15A** and **15B**

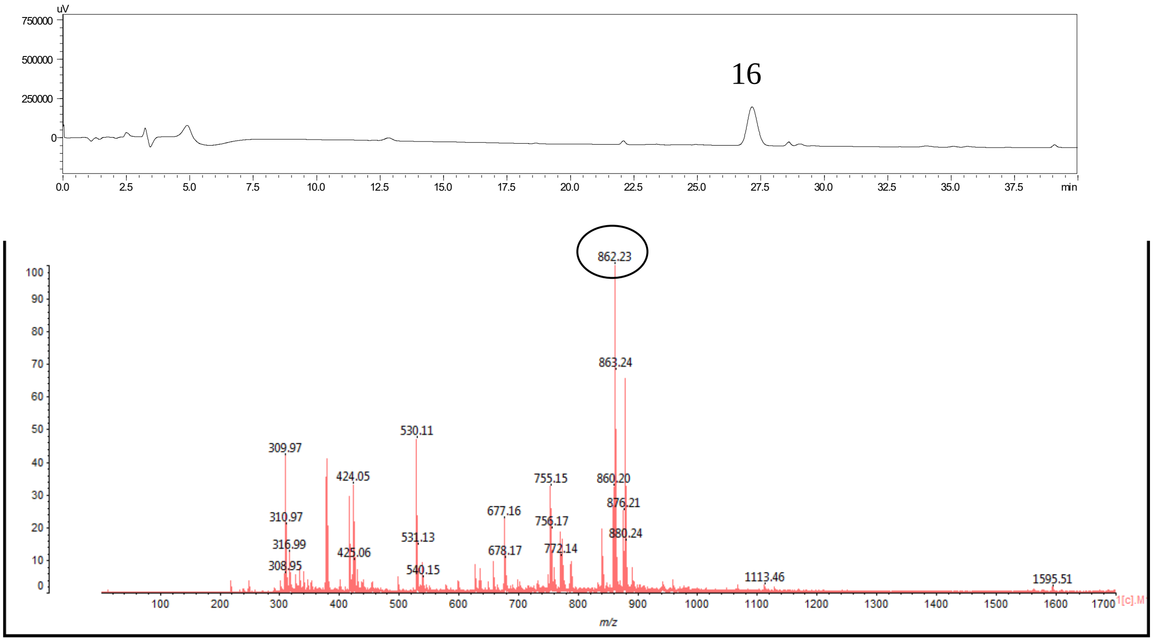

Figure S33. HPLC chromatogram and MALDI-TOF spectrum of peptoid **16**

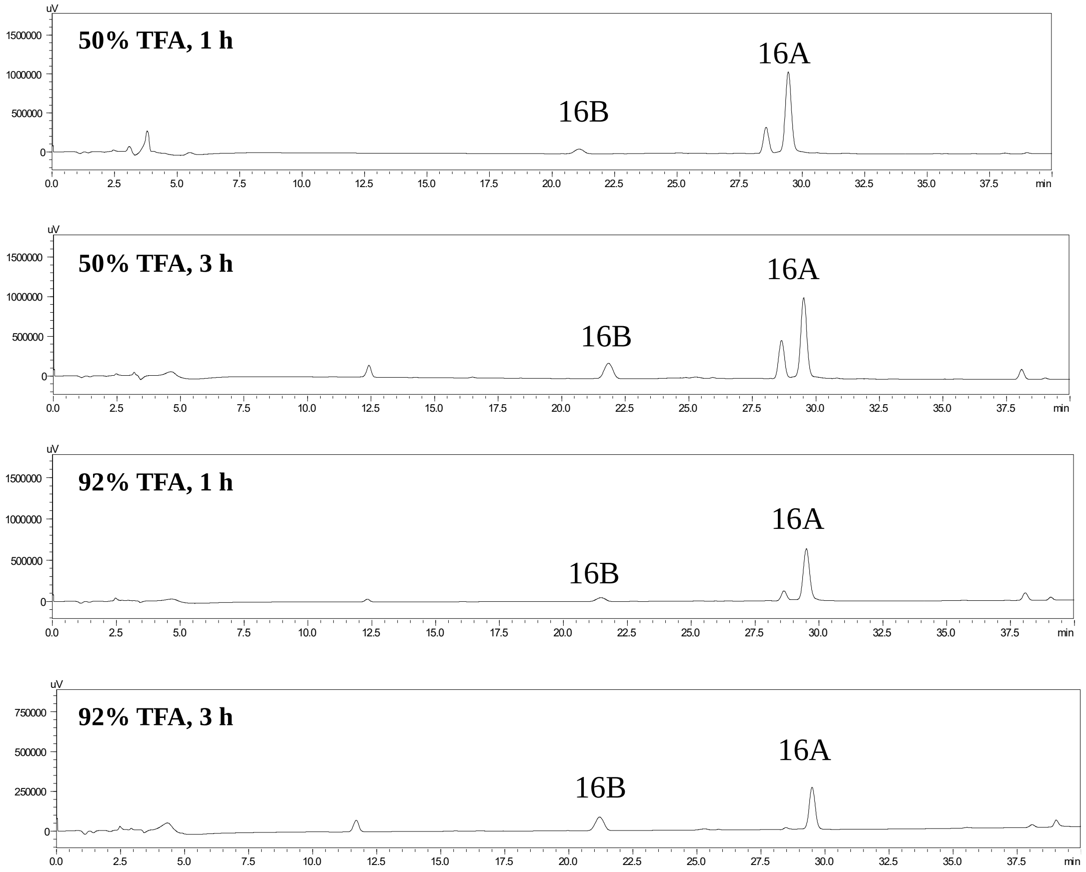

Figure S34. HPLC chromatograms after TFA-assisted truncation of peptoid **16A’**

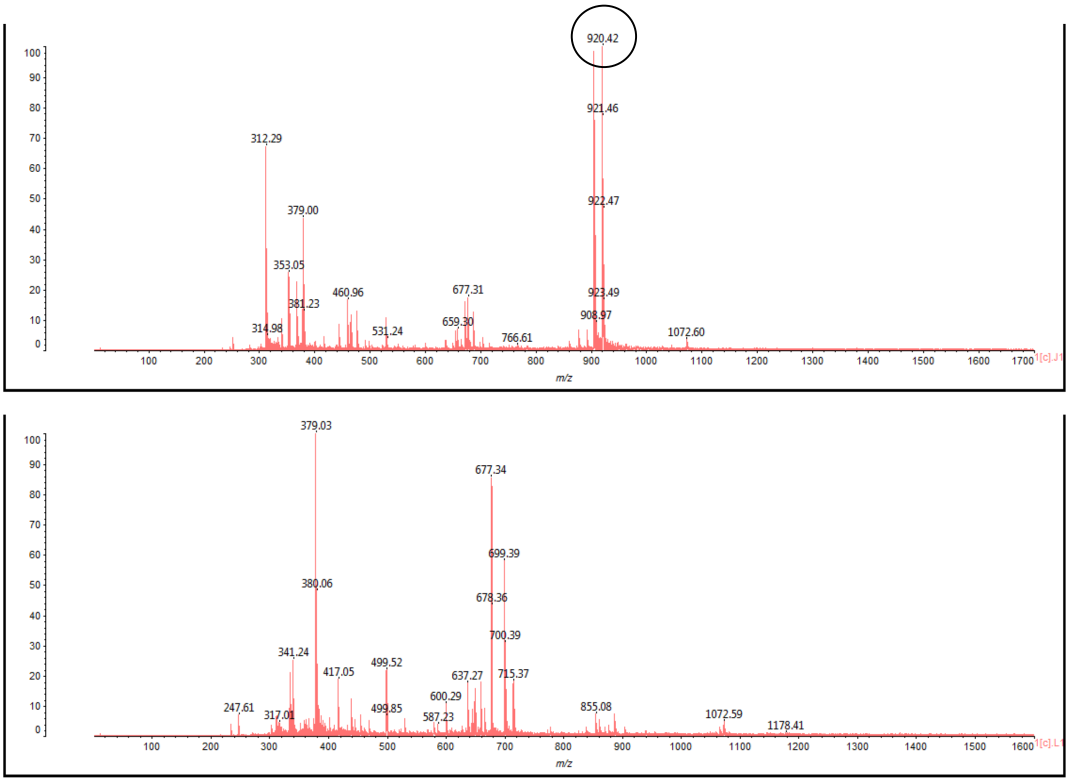

Figure S35. MALDI-TOF spectra of peptoids **16A** and **16B**

(b)

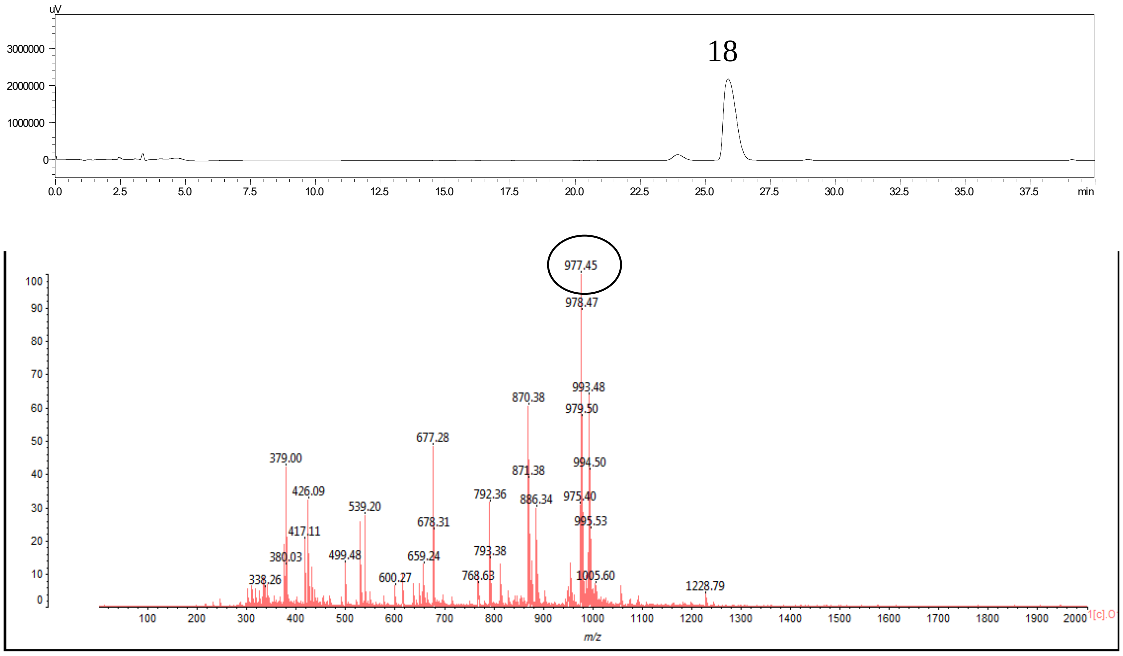

Figure S36. HPLC chromatogram and MALDI-TOF spectrum of peptoid **17**

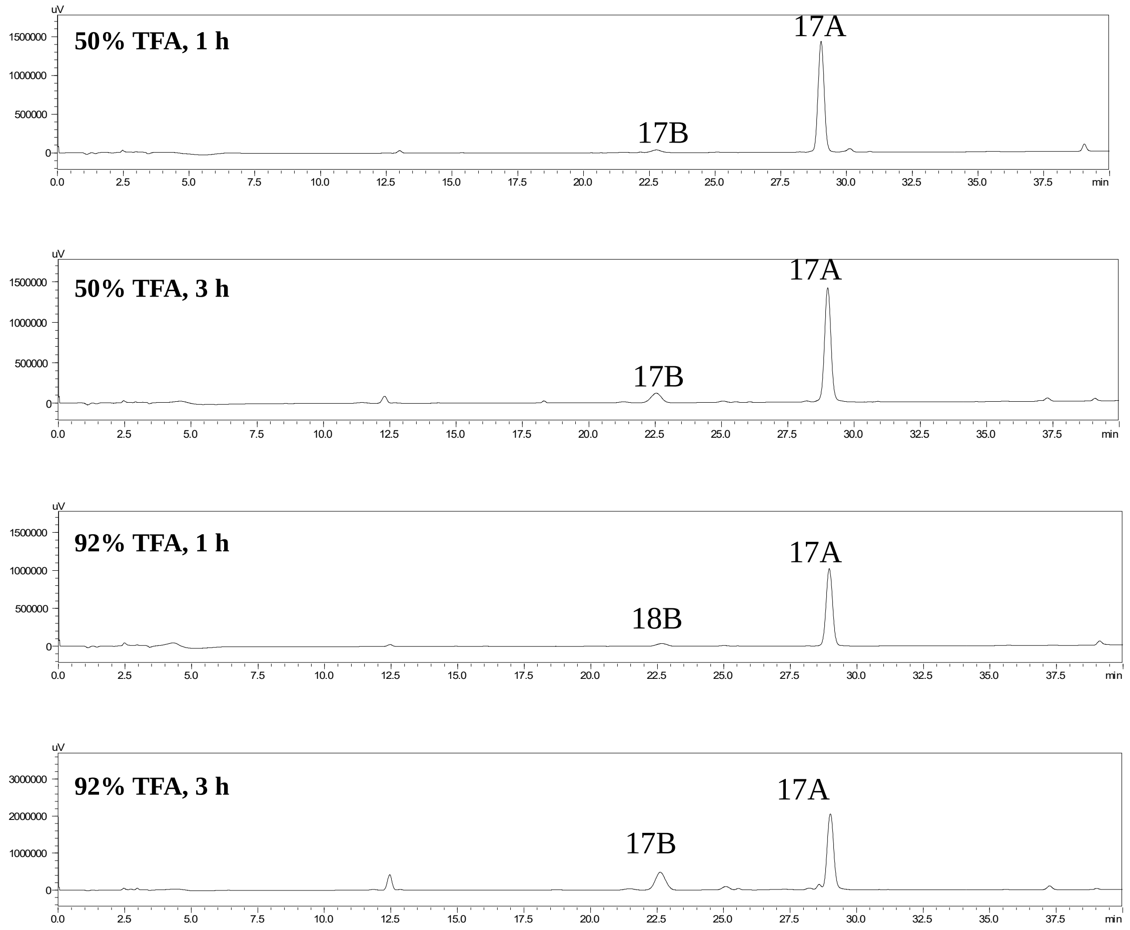

Figure S37. HPLC chromatograms after TFA-assisted truncation of peptoid **17A'**

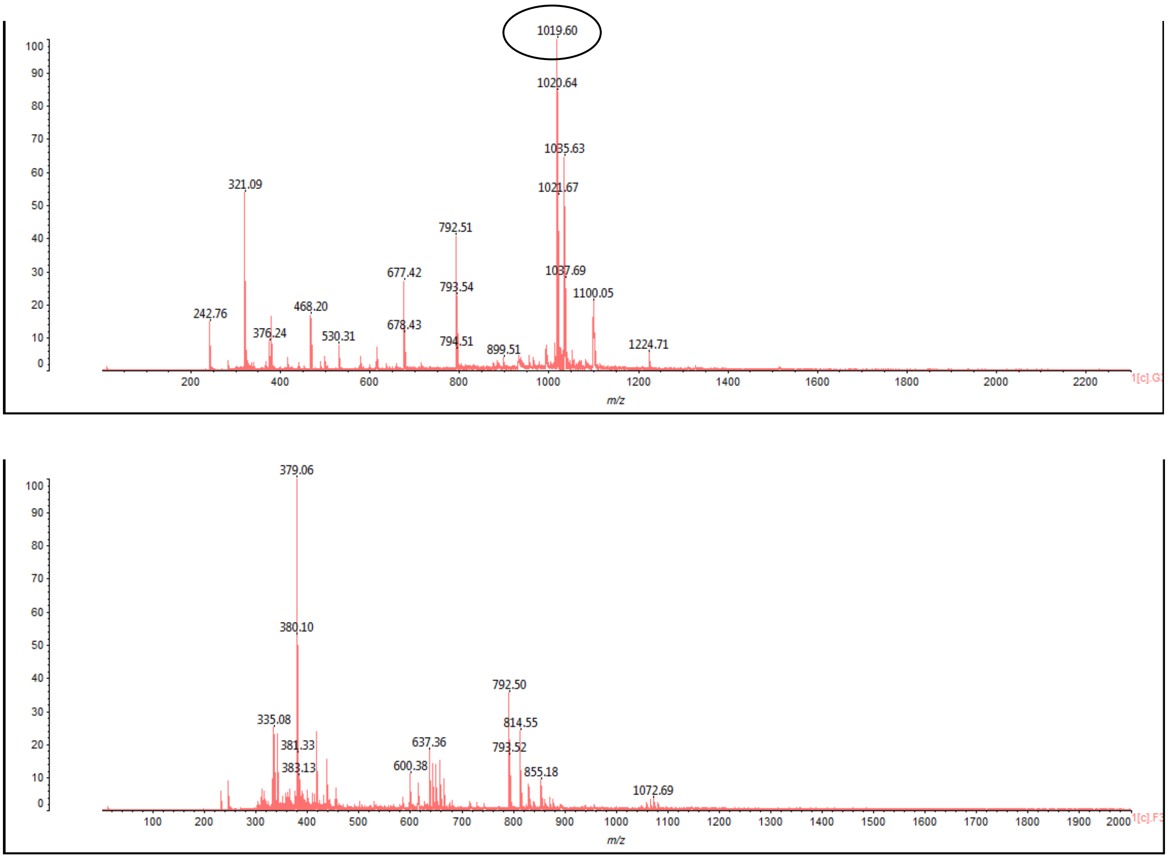

Figure S38. MALDI-TOF spectra of peptoids **17A** and **17B**

Table S5. Comparison of *N*-branched and *N*-unbranched alkyl side chains on TFA-assisted truncation of peptoids

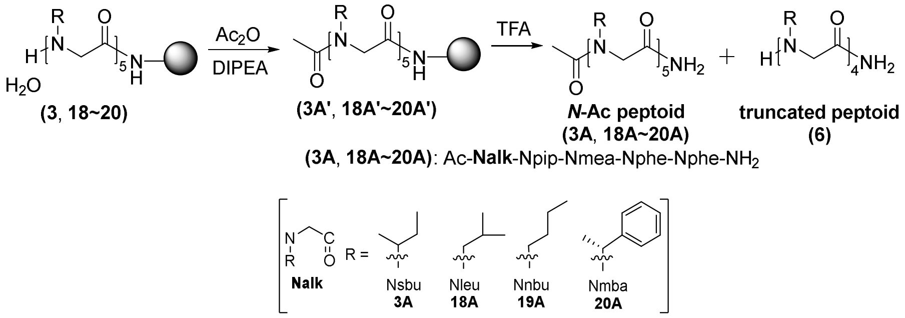

Entry	Peptoid	Truncated peptoid (**6**) (%) [a]			
		Cond. A [b]		Cond. B [c]	
		1 h	3 h	1 h	3 h
1	**3A′**	100	-	100	-
2	**18A′**	65.5	84.0	69.8	85.8
3	**19A′**	44.0	76.7	69.4	78.5
4	**20A′**	70.0	86.7	86.9	100

[a] Relative yields of peptoids were determined by HPLC analysis.
[b] Reaction conditions: 50% TFA/3% TIS/5% H_2O/42% CH_2Cl_2.
[c] Reaction conditions: 92% TFA/3% TIS/5% H_2O.

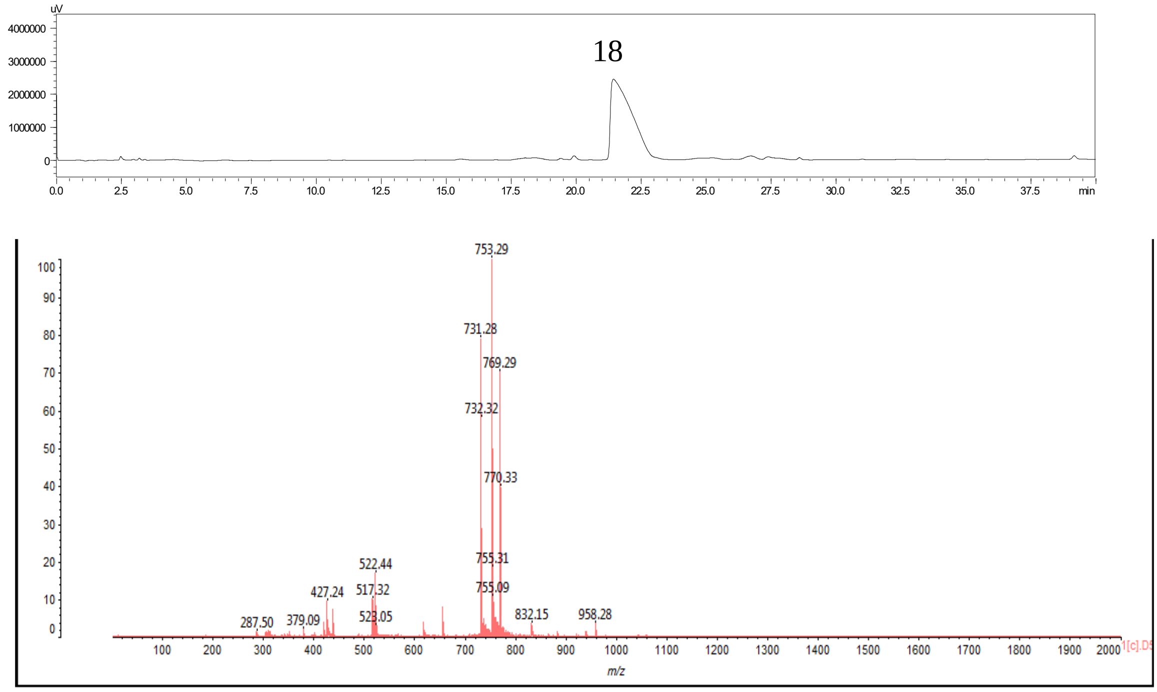

Figure S39. HPLC chromatogram and MALDI-TOF spectrum of peptoid **18**

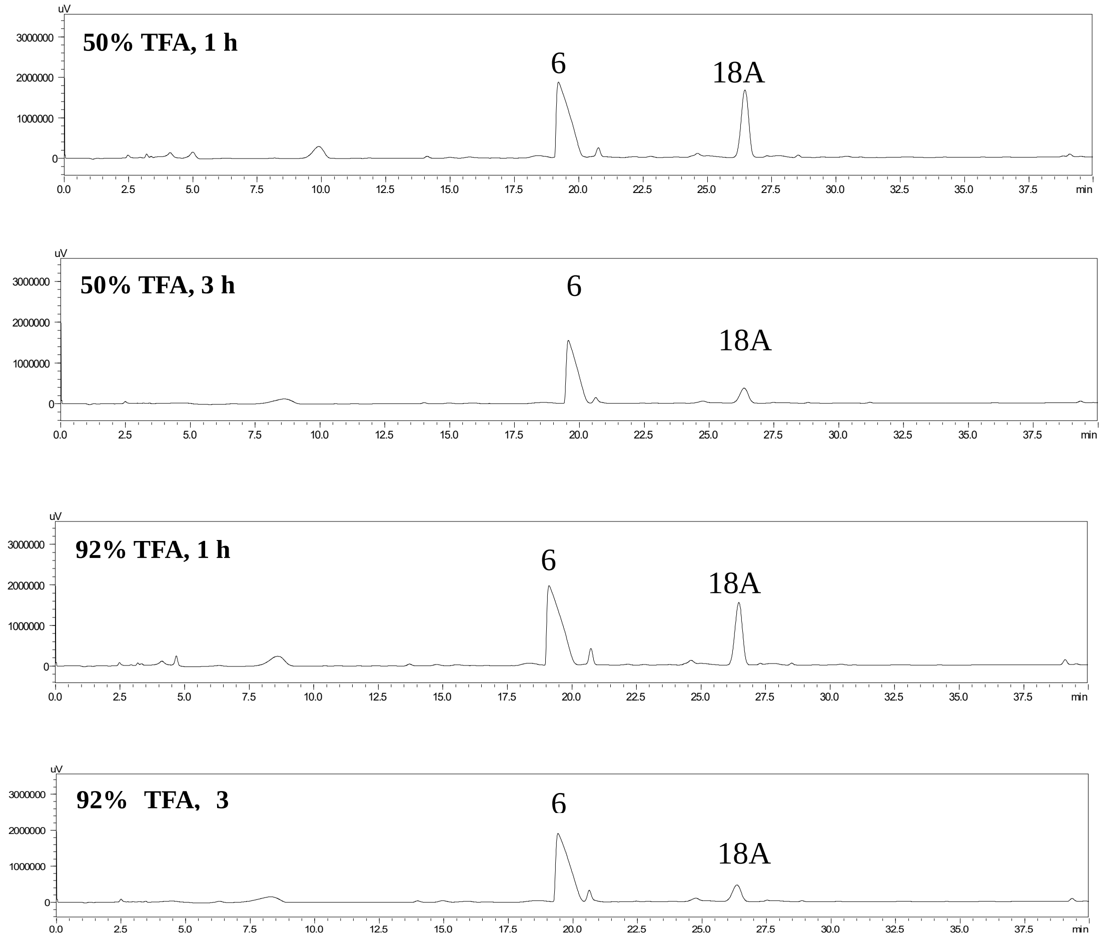

Figure S40. HPLC chromatograms after TFA-assisted truncation of peptoid **18A'**

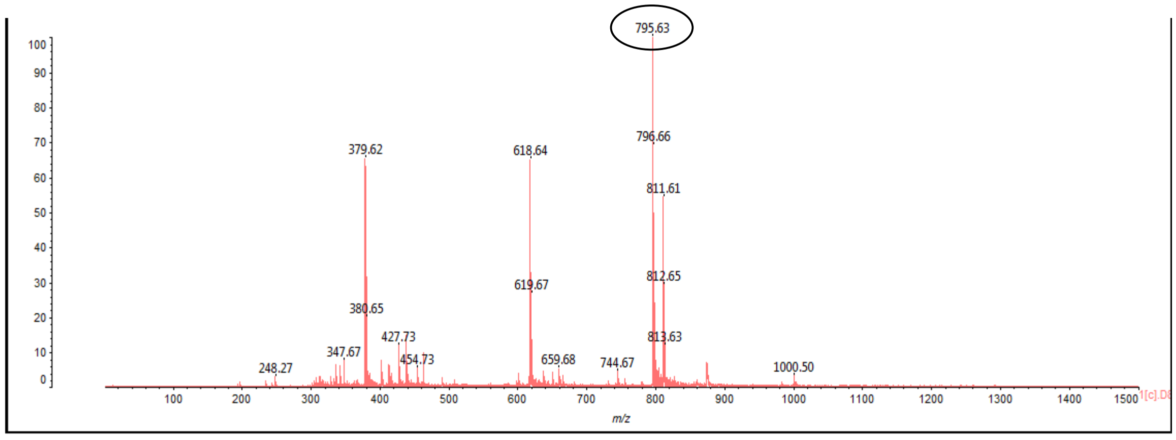

Figure S41. MALDI-TOF spectrum of peptoid **18A**

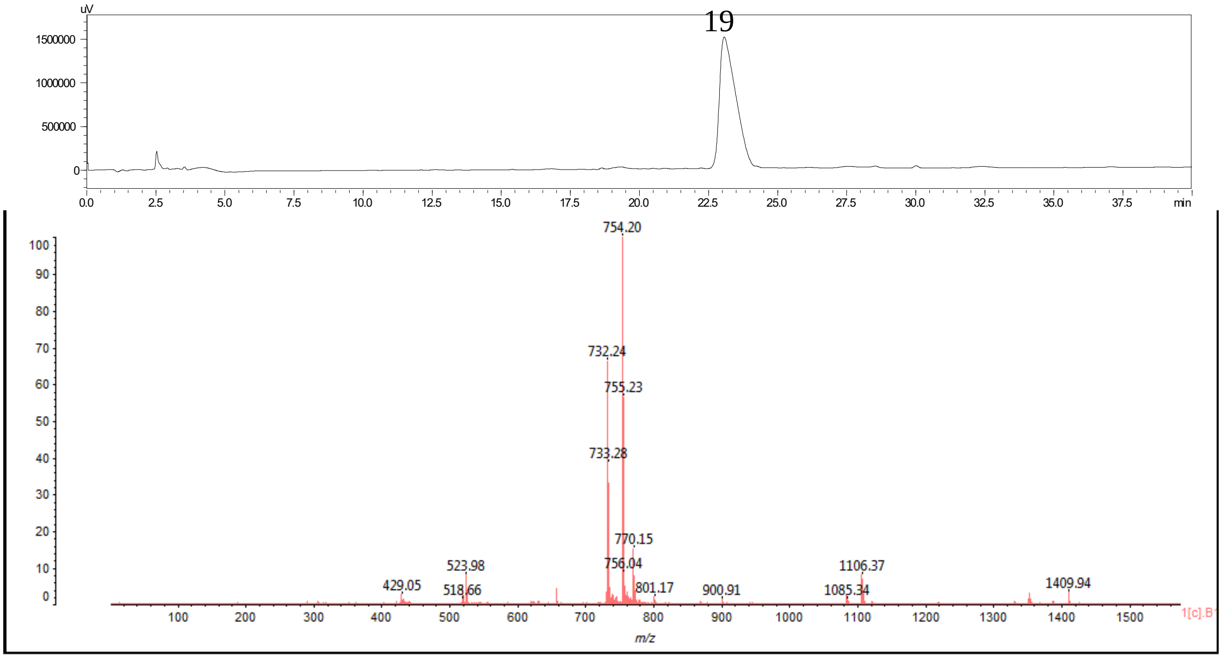

Figure S42. HPLC chromatogram and MALDI-TOF spectrum of peptoid **19**

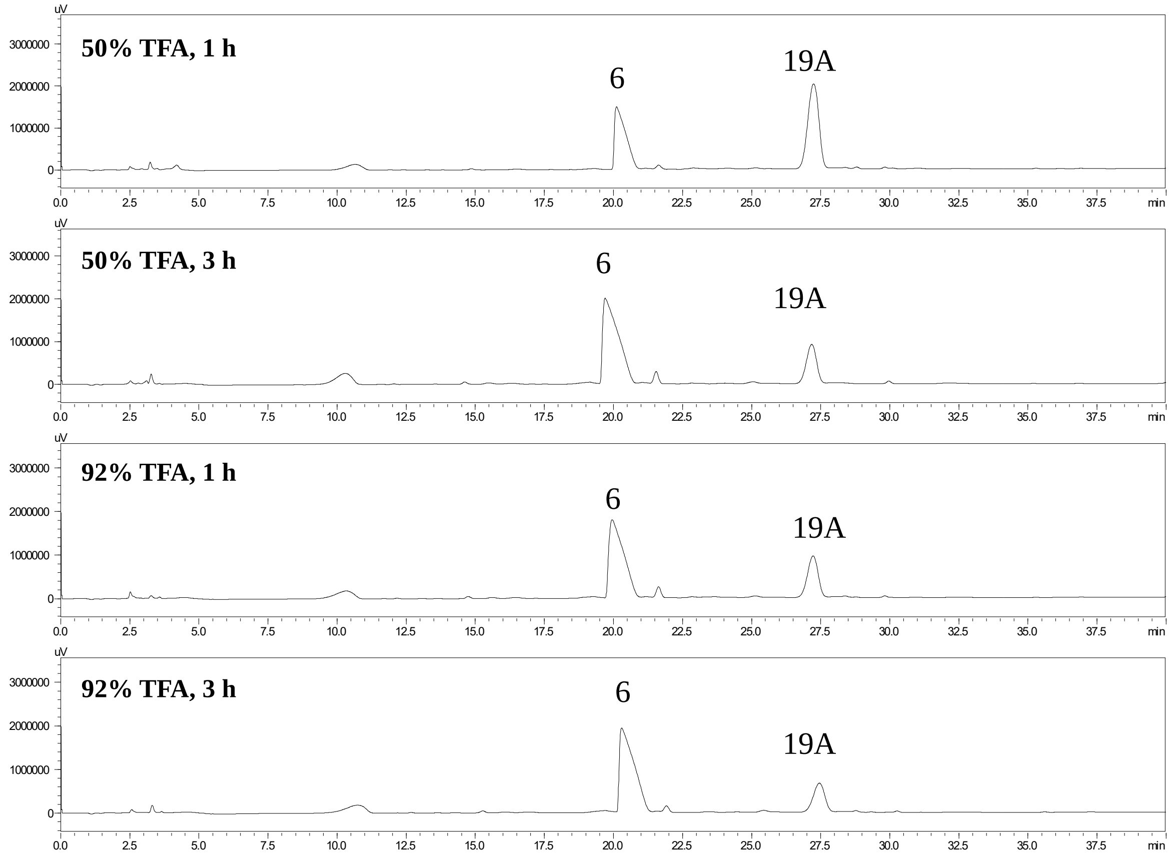

Figure S43. HPLC chromatograms after TFA-assisted truncation of peptoid **19A'**

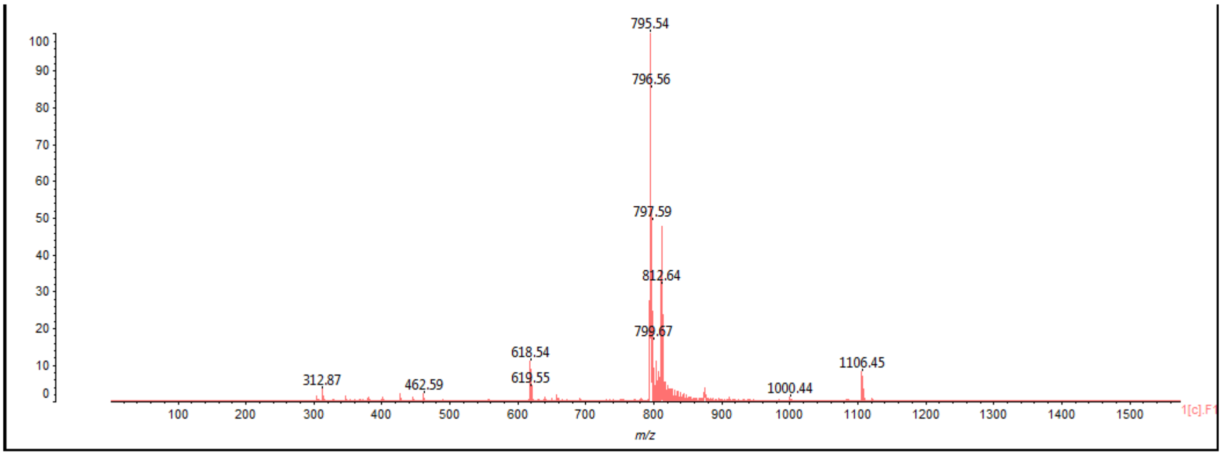

Figure S44. MALDI-TOF spectrum of peptoid **19A**

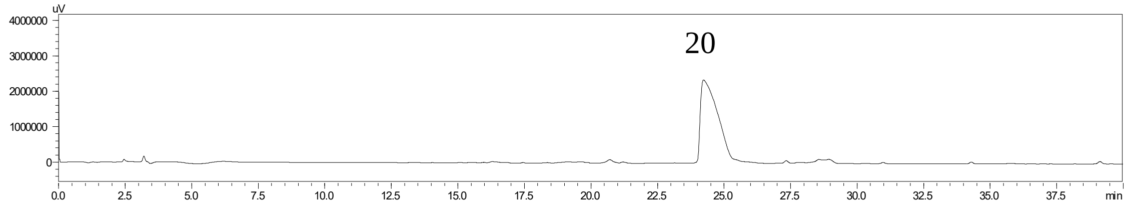

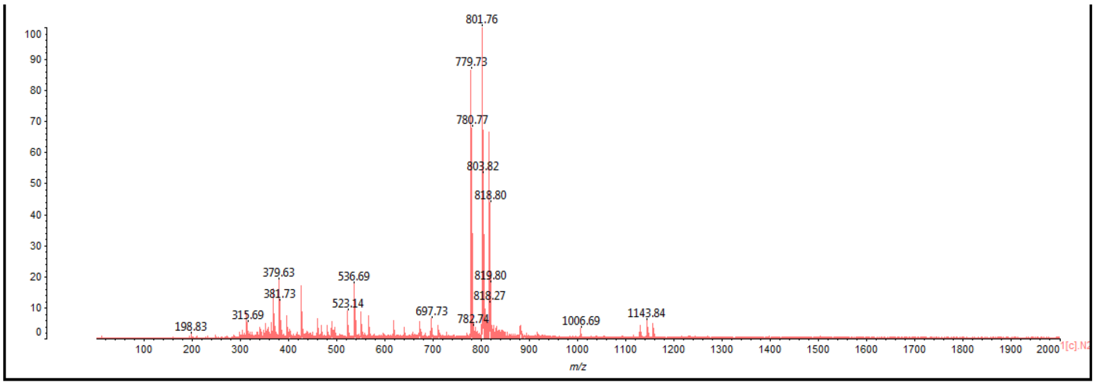

Figure S45. HPLC chromatogram and MALDI-TOF spectrum of peptoid **20**

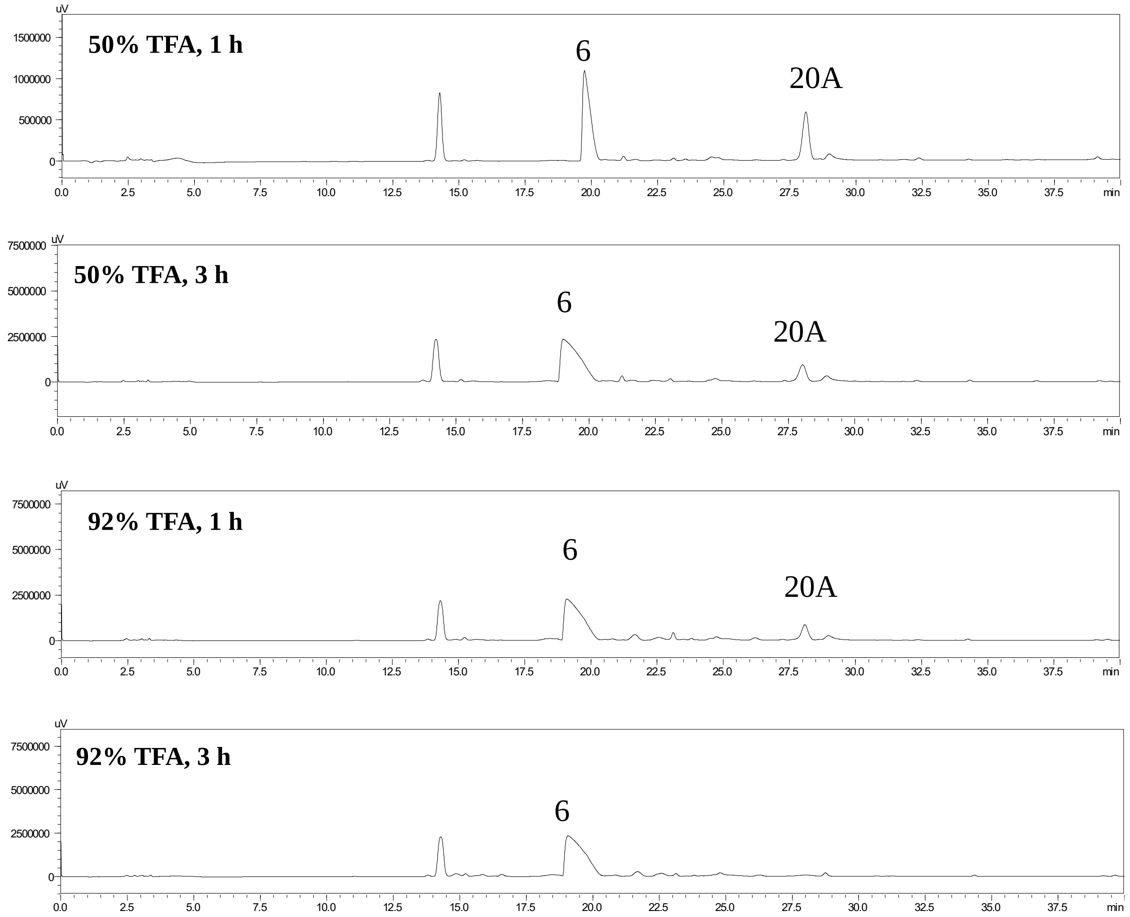

Figure S46. HPLC chromatograms after TFA-assisted truncation of peptoid **20A'**

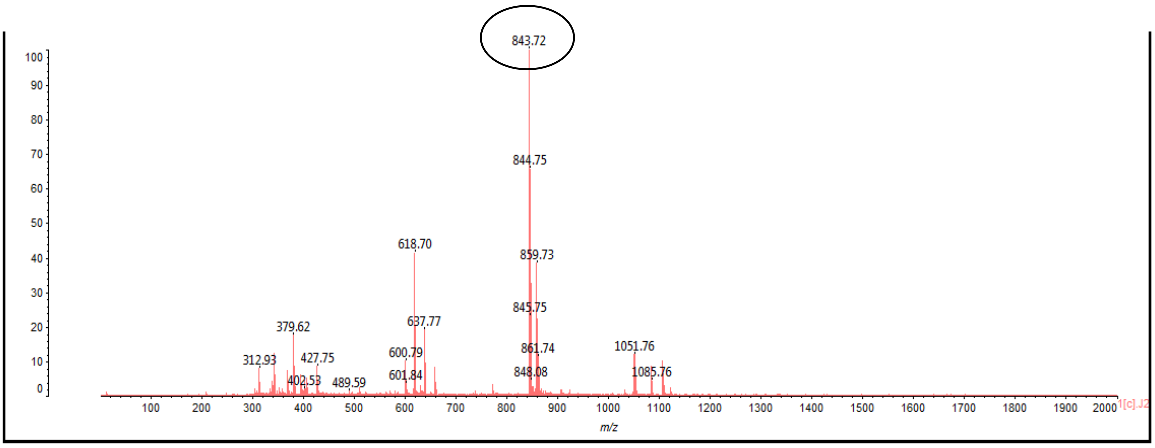

Figure S47. (c) HPLC spectrum and (d) MALDI-TOF spectrum of Linear peptoid **20A**
